# Central Insulin-like Growth Factor-1 Treatment Enhances Working and Reference Memory by Reducing Neuroinflammation and Amyloid Beta Deposition in a Rat Model of Sporadic Alzheimer’s Disease

**DOI:** 10.3390/ph18040527

**Published:** 2025-04-04

**Authors:** Joanna Dunacka, Beata Grembecka, Irena Majkutewicz, Danuta Wrona

**Affiliations:** Department of Animal and Human Physiology, Faculty of Biology, University of Gdansk, 59 Wita Stwosza Str, 80-308 Gdansk, Poland; joanna.dunacka@ug.edu.pl (J.D.); beata.grembecka@ug.edu.pl (B.G.); irena.majkutewicz@ug.edu.pl (I.M.)

**Keywords:** IGF-1, insulin signaling, sporadic Alzheimer’s disease, spatial memory, neuroinflammation, amyloid-β, streptozotocin, rat

## Abstract

**Background/Objectives**: Brain insulin resistance is a potential causal factor for dementia in Alzheimer’s disease (AD). Insulin-like growth factor-1 (IGF-1), a neurotrophin, plays a key role in central insulin signaling and neuroprotection. Intracerebrovenitricular (ICV) administration of streptozotocin (STZ) disrupts insulin signal transduction, leading to brain insulin resistance, which may mimic the early pathophysiological changes in sporadic AD (sAD). In this study, we investigated whether restoring insulin signaling through ICV injection of IGF-1 could ameliorate spatial memory deficits during sAD progression in a rat model induced by ICV STZ injection. **Methods**: Male Wistar rats (n = 40) were subjected to double ICV injections of STZ (0.75 mg/kg/ventricle, days 2 and 4) and IGF-1 (1 μg/single injection, days 1 and 3), and placed at the Morris water maze (MWM) at baseline, 7, 45 and 90 days after injections. Reference (days 1–3 and day 4 MWM)) and working (days 5–8 MWM) memory, microglia activation (CD68^+^ cells), and amyloid β (Aβ) deposition (immunohistochemistry) were measured. **Results**: We found that ICVIGF-1 administration protected working memory demonstrated as (1) reduced latency to reach the platform, and reduced swimming distance in trials 3 (*p* < 0.05) and 4 (*p* < 0.01) on days 45 and 90 post-injection and (2) a short-term (up to 45 days post-injection) enhancement of reference memory, manifested by a reduction in swimming distance and latency (*p* < 0.05). Furthermore, IGF-1 treatment reduced neuroinflammation in CA2 (*p* < 0.05) and Aβ deposition in CA1 (*p* < 0.01) of the hippocampus. **Conclusions**: Central IGF-1 attenuates spatial memory deficits in the ICVSTZ-induced sAD model by reducing neuroinflammation and Aβ accumulation in the hippocampus.

## 1. Introduction

Glucose metabolism, including insulin-like growth factor-1 (IGF-1) signaling, plays a crucial role in the central regulation of metabolism. However, dysfunction of insulin signaling in the aging brain leads to abnormal energy metabolism, brain insulin resistance, and dementia, as observed in Alzheimer’s disease (AD) [1]. The IGF-1 signaling pathway is critical for the central nervous system’s development, metabolism, repair, cognition, and emotion regulation [2]. IGF-1 in the brain has neuroprotective, anti-apoptotic, and neurotrophic properties [1], including increasing the survival of cholinergic neurons involved in the consolidation of memory traces [1] and promoting the conversion of microglia to an anti-inflammatory M2 phenotype, thereby repairing damaged neural tissue in AD [3] or after stroke [4] by inhibiting neuroinflammation. This neurotrophin has protective properties against amyloid-β (Aβ)-induced toxicity in hippocampal cells [1]. Furthermore, the cooperation of insulin and IGF-I is needed to recover neuronal activity after hypoglycemia [2].

Insulin plays multiple neuronal roles, including regulating synaptic plasticity and synapse density [5,6,7]. Emerging evidence suggests that disruptions in the insulin/IGF-1/growth hormone axis, characteristic of diabetes, contribute to excessive Aβ production and neurodegenerative processes, potentially driving AD pathology. Hyperinsulinemia associated with type 2 diabetes leads to decreased insulin signaling in the brain [8]; thus, the brain in AD is in a state of insulin resistance [9], and AD has been referred to as “type 3 diabetes” [10,11]. Furthermore, loss of insulin receptors in the brain leads to increased levels of hyperphosphorylated Tau [12,13], which is prone to form neurofibrillary tangles in neurons [14]. Molecular findings suggest that insulin resistance and dysregulated IGF-1 signaling promote atherosclerosis and a pro-inflammatory state [13]. It suggests that type 2 mellitus and AD, in particular sporadic AD (sAD), share common risk factors, and molecular mechanisms, including impairment of insulin/IGF—1 signaling, and thus potentially common therapeutic targets [15].

Recent preclinical and clinical evidence indicates that central IGF-1, apart from regulating growth and development, prevents neuronal death mediated by amyloidogenesis, cerebral glucose deprivation, neuroinflammation, and apoptosis through modulation of phosphoinositide 3-kinase-protein kinase B (PI3K), mammalian target of rapamycin and mitogen-activated protein kinase [16]. Acute administration of IGF-1 centrally can restore whole-body glucose homeostasis in old insulin-resistant animals [17,18]. IGF-1, as a regulator of oxidative stress responses, plays a regulatory role in the emergence of progressive neurodegenerative and neuroinflammatory conditions. However, the role of IGF-1 in the aging brain is complex and debated, due to its conflicting positive and negative effects [11,19]. In certain contexts, reduced IGF-1 signaling can enhance survival by limiting the production of reactive oxygen species and age-related neurodegenerative disorders [20,21]. Conversely, a deficiency in IGF-1 has been linked to cognitive impairments and a higher risk of poor cognitive performance in humans [19,22].

Intracerebroventricular (ICV) injection of streptozotocin (STZ) is a widely used animal model for mimicking dementia associated with sAD [23]. ICVSTZ injections alter the functioning of enzymes involved in cerebral glucose metabolism [24] and disrupt the insulin/IGF pathway simulating some of the cerebral alterations observed in AD [25]. ICVSTZ administration also induces neuroinflammation and oxidative stress [26], which are major characteristic features of AD and other dementias, and thus affects neurogenesis [27]. One of the early events observed during sporadic AD (sAD) was that ICVSTZ injection induces brain insulin receptor dysfunction [15,25] leading to a resistant brain state. Therefore, in this study, we investigated the effect of ICVIGF-1 administration, as a potential protective mechanism to counteract the ICVSTZ co-injection-induced insulin resistance state of the brain and changes in behavioral activity associated with reference and working memory (Morris Water Maze; MWM), neuroinflammation measured by number of activated microglia cells (CD68^+^), and Aβ deposition in the brain. To test the potential protective effect of IGF-1 on sAD progression in the rat model, we examined these parameters before ICV injection of IGF-1 and STZ (baseline) and after 7, 45, and 90 days of disease progression. We used the same procedure to induce the sAD model, as in our previous studies (injections of ICVSTZ at a cumulative dose of 3 mg/kg administered twice on days 2 and 4) [28,29,30,31,32]. Our findings suggest that enhancing central insulin signaling via IGF-1 can improve cognitive functions in the rat model of sAD induced by ICV administration of STZ. This suggests the potential role of IGF-1 as a novel therapeutic target, also in combination with insulin, in the treatment of dementia in sporadic Alzheimer’s disease.

## 2. Results

### 2.1. Spatial Memory Impairments in the Morris Water Maze (MWM) Test

#### 2.1.1. Reference Memory in the Morris Water Maze (MWM) Test

**Figure 1:**
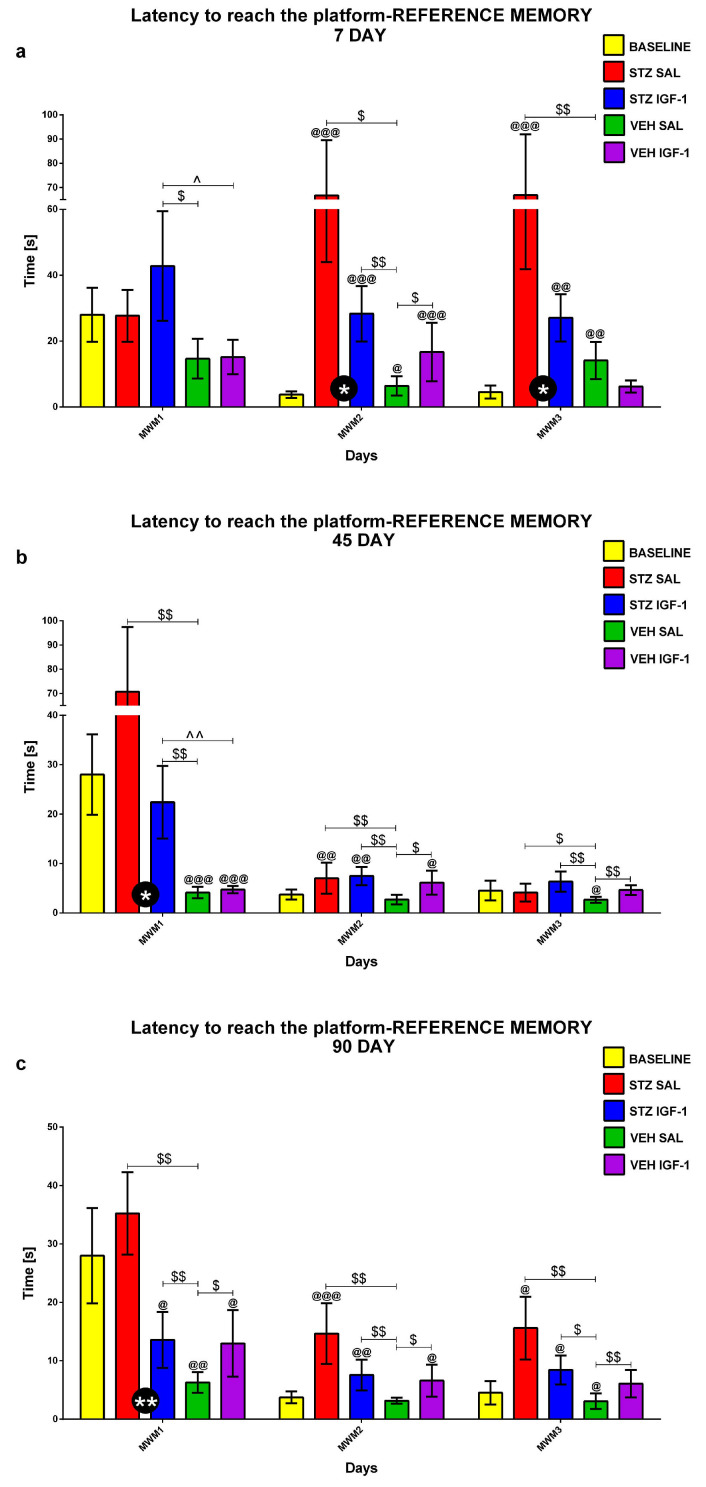
Reference memory in the Morris water maze (MWM) test during 3 consecutive days (MWM1-MWM3, Trial 1) measured as latency to reach the platform in rats before injection (BASELINE, n = 40) and 7 (**a**), 45 (**b**), and 90 (**c**) days after intracerebroventricular injection of streptozotocin (STZ) and saline (SAL) (STZSAL, n = 10) or STZ and insulin-like growth factor-1 (IGF-1) (STZIGF-1, n = 10) or citrate buffer (VEH) and SAL (VEHSAL, n = 10) or VEH and IGF-1 (VEHIGF-1, n = 10). Explanations: Data are presented as mean ± SD and were analyzed using Mann–Whitney-U test; * in a circle—*p* < 0.05, ** in a circle—*p* < 0.01 difference between STZSAL and STZIGF-1 group; $ above the line between the bars—*p* < 0.05, $$ above the line between the bars—*p* < 0.01 difference vs. VEH SAL group; ^ above the line between the bars—*p* < 0.05, ^^ above the line between the bars—*p* < 0.01 difference vs. VEH IGF-1 group; @—*p* < 0.05, @@—*p* < 0.01, @@@—*p* < 0.001 difference vs. baseline.

**Figure 2:**
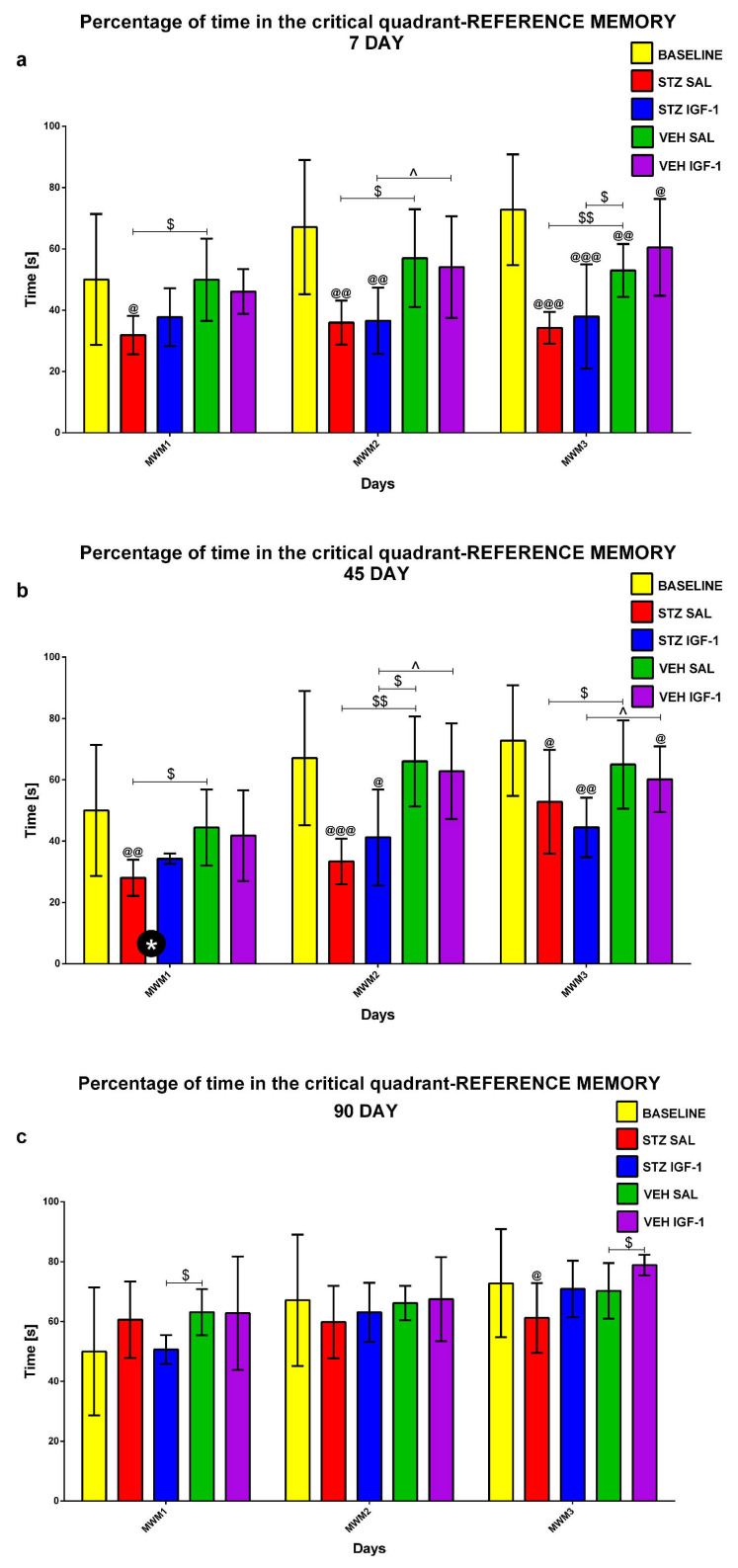
Reference memory in the Morris water maze (MWM) test during 3 consecutive days (MWM1-MWM3, Trial 1) measured as percentage of time spent in the critical quadrant in rats before injection (BASELINE, n = 40) and 7 (**a**), 45 (**b**), and 90 (**c**) days after intracerebroventricular injection of streptozotocin (STZ) and saline (SAL) (STZSAL, n = 10) or STZ and insulin-like growth factor-1 (IGF-1) (STZIGF-1, n = 10) or citrate buffer (VEH) and SAL (VEHSAL, n = 10) or VEH and IGF-1 (VEHIGF-1, n = 10). Explanations: Data are presented as mean ± SD and were analyzed using Mann–Whitney-U test; * in a circle—*p* < 0.05, difference between STZSAL and STZIGF-1 group; $ above the line between the bars—*p* < 0.05, $$ above the line between the bars—*p* < 0.01 difference vs. VEH SAL group; ^ above the line between the bars—*p* < 0.05, difference vs. VEHIGF-1 group; @—*p* < 0.05, @@—*p* < 0.01, @@@—*p* < 0.001 difference vs. baseline.

**Figure 3:**
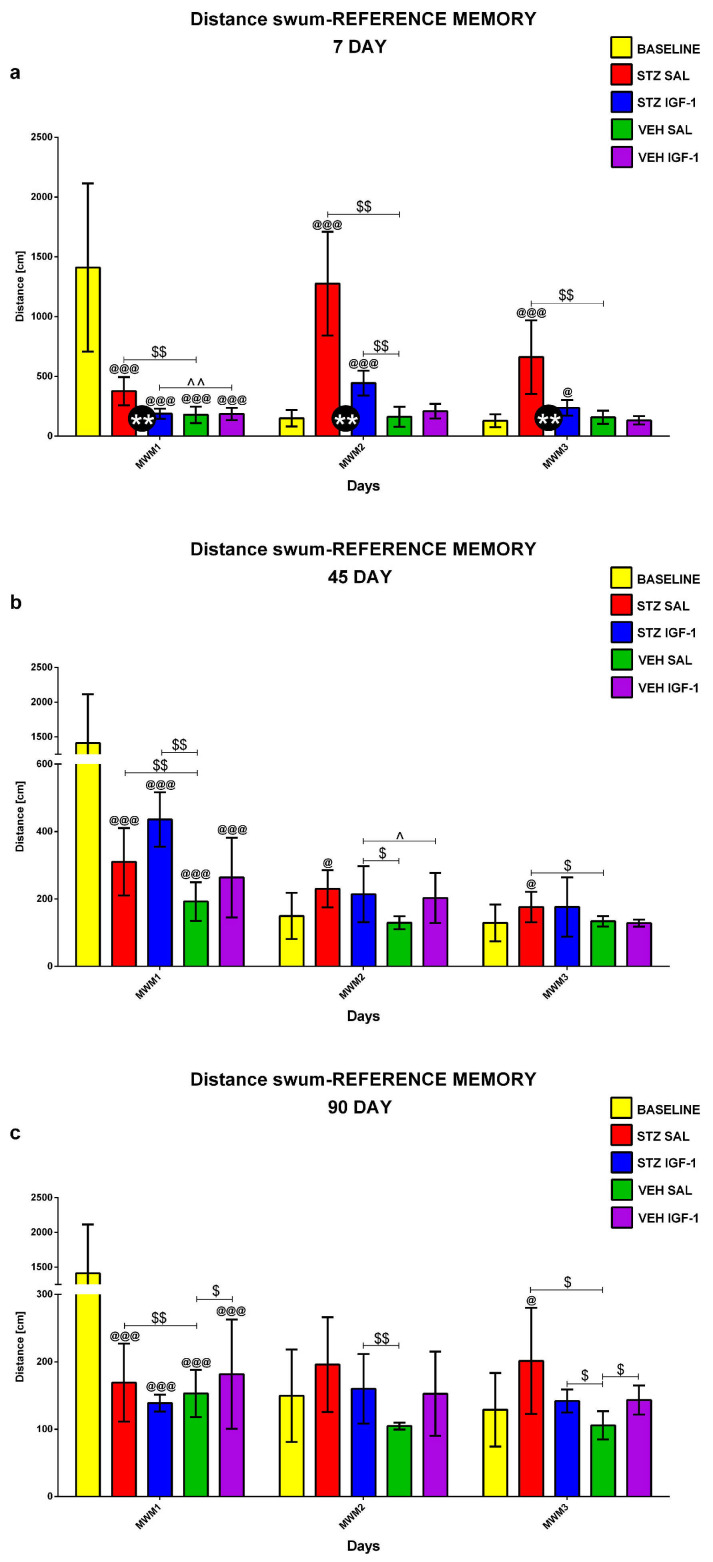
Reference memory in the Morris water maze (MWM) test during 3 consecutive days (MWM1-MWM3, Trial 1) measured as total distance swum in rats before injection (BASELINE, n = 40) and 7 (**a**), 45 (**b**), and 90 (**c**) days after intracerebroventricular injection of streptozotocin (STZ) and saline (SAL) (STZSAL, n = 10) or STZ and insulin-like growth factor-1 (IGF-1) (STZIGF-1, n = 10) or citrate buffer (VEH) and SAL (VEHSAL, n = 10) or VEH and IGF-1 (VEHIGF-1, n = 10). Explanations: Data are presented as mean ± SD and were analyzed using Mann–Whitney-U test; ** in a circle—*p* < 0.01, difference between STZSAL and STZIGF-1 group; $ above the line between the bars—*p* < 0.05, $$ above the line between the bars—*p* < 0.01 difference vs. VEH SAL group; ^ above the line between the bars—*p* < 0.05, ^^ above the line between the bars—*p* < 0.01 difference vs. VEH IGF-1 group; @—*p* < 0.05, @@@—*p* < 0.001 difference vs. baseline.

Reference memory in the MWM during 3 consecutive days (MWM1-MWM3, 4 Trials/day) and on the day without the platform (probe test, 1 Trial) was investigated at the baseline conditions (before injection) and starting from days 7, 45, and 90 after injection. Figure 1, Figure 2 and Figure 3 show the results for behavioral activity during Trial 1 measured as latency to reach the platform (Figure 1), time spent in the critical quadrant (Figure 2), and total distance swum (Figure 3) within the STZSAL, STZIGF-1, VEHSAL, and VEHIGF-1 groups in comparison with the baseline (before injection).

Compared to STZSAL rats, the latency to reach the platform was significantly shorter in the STZIGF-1 group on days 8–9 (corresponding to MWM days 2 and 3, *p* < 0.05; Figure 1a), day 45 (MWM day 1, *p* < 0.05; Figure 1b), and day 90 post-injection (MWM day 1, *p* < 0.01; Figure 1c). At days 91 and 92 post-injection (days 2 and 3 of the MWM) in the STZIGF-1 group, the latency was shorter compared to the STZSAL group, but the difference did not reach statistical significance. In the STZIGF-1 animals, the time spent in the critical quadrant was significantly longer 45 days after injection (Figure 2b; *p* < 0.05) and the total distance swum was shorter at 7–9 days post-injection (Figure 3a; days 1–3 of the MWM, *p* < 0.01), compared to the STZSAL group. At both 45 and 90 days post-injection, there were no significant differences in swimming distance between the STZIGF-1 and STZSAL groups, although a trend toward reduced swimming distance was observed in STZIGF-1 animals.

In addition, there was no significant difference between the STZIGF-1 and control VEHIGF-1 groups in the latency to reach the platform (except for days 7 and 45 post-injection, Figure 1a,b), in the time spent in the critical quadrant (except for days 46 and 47 post-injection, Figure 2b) and in the swimming distance (except for days 7 and 46 post-injection, Figure 3a,b). However, despite significantly shorter latency to reach the platform (Figure 1) and swimming distance (Figure 3) during Trial 1 of 3 consecutive days of the MWM compared to the STZSAL group, these parameters were still worsened in STZIGF-1 animals, particularly at days 45 and 90 after injection, compared to the control VEHSAL group.

#### 2.1.2. Reference Memory Performance in the Probe Test on Day 4 of the Morris Water Maze (MWM) Test

**Figure 4:**
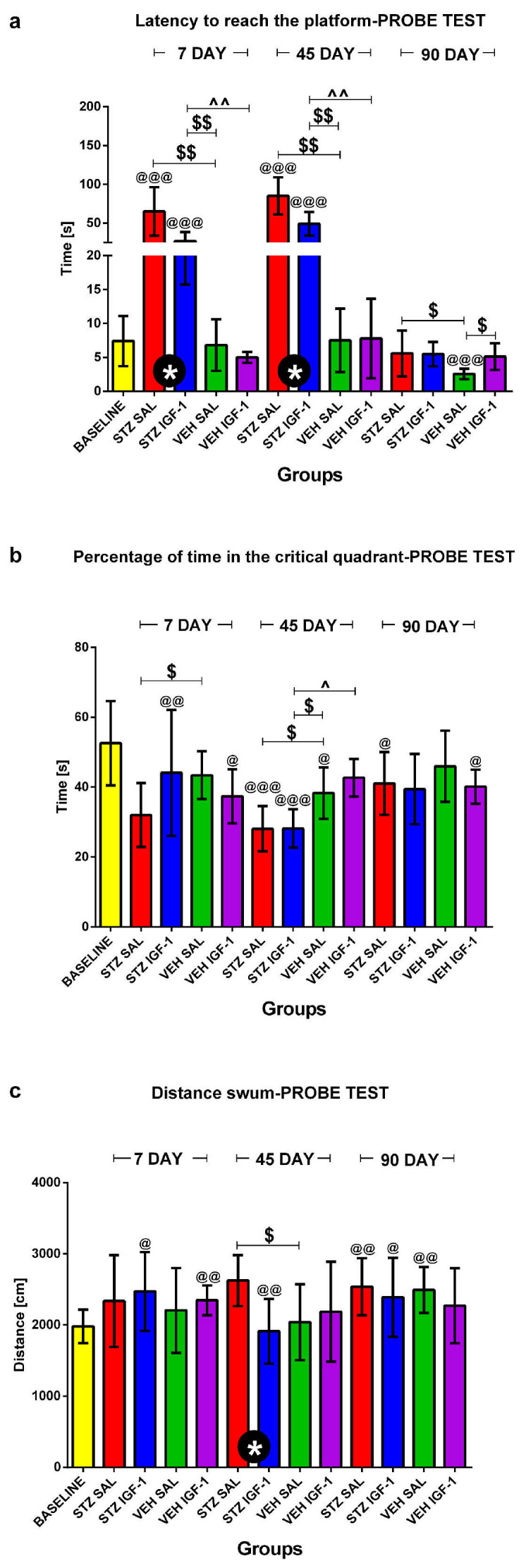
Reference memory performance in the Morris water maze (MWM) test measured as latency to reach the platform (**a**), the percentage of time in the critical quadrant (**b**), and the total distance swum (**c**) on a day without the platform (probe test) in rats before injection (BASELINE, n = 40) and 7, 45 and 90 days after intracerebroventricular injection of streptozotocin (STZ) and saline (SAL) (STZSAL, n = 10) or STZ and insulin-like growth factor-1 (IGF-1) (STZIGF-1, n = 10) or citrate buffer (VEH) and SAL (VEHSAL, n = 10) or VEH and IGF-1 (VEHIGF-1, n = 10). Explanations: Data are presented as mean ± SD and were analyzed using Mann–Whitney-U test; * in a circle—*p* < 0.05 difference between STZSAL and STZIGF-1 group; $ above the line between the bars -*p* < 0.05, $$ above the line between the bars—*p* < 0.01 difference vs. VEHSAL; ^ above the line between the bars—*p* < 0.05, ^^ above the line between the bars—*p* < 0.01 difference vs. VEHIGF-1; @—*p* < 0.05, @@—*p* < 0.01, @@@—*p* < 0.001 difference vs. baseline.

The reference memory during the probe test is presented in Figure 4 and shows reference memory performance during a single trial on day 4 of the MWM when the platform was removed. As shown in Figure 4a, the latency to reach the platform was significantly (*p* < 0.05) shorter in the STZIGF-1 group compared with STZSAL animals 7 and 45 days after injection. However, the latency was significantly longer in both STZSAL and STZIGF-1 animals compared to the controls: VEHSAL and VEHIGF-1 groups at days 7 (*p* < 0.01) and 45 (*p* < 0.01) after STZ injection. In addition, there was a significantly (*p* < 0.05) reduced total swimming distance in the STZIGF-1 animals, compared to the STZSAL group at day 45 post-injection (Figure 4c and Figure S1). On the other hand, there were no significant differences in the percentage of time spent in the critical quadrant (Figure 4b) between the STZIGF-1 and STZSAL groups 7, 45, and 90 days after injection.

#### 2.1.3. Working Memory During Trials 1–4 of Consecutive Days 5–8 of the Morris Water Maze (MWM) Test

**Figure 5:**
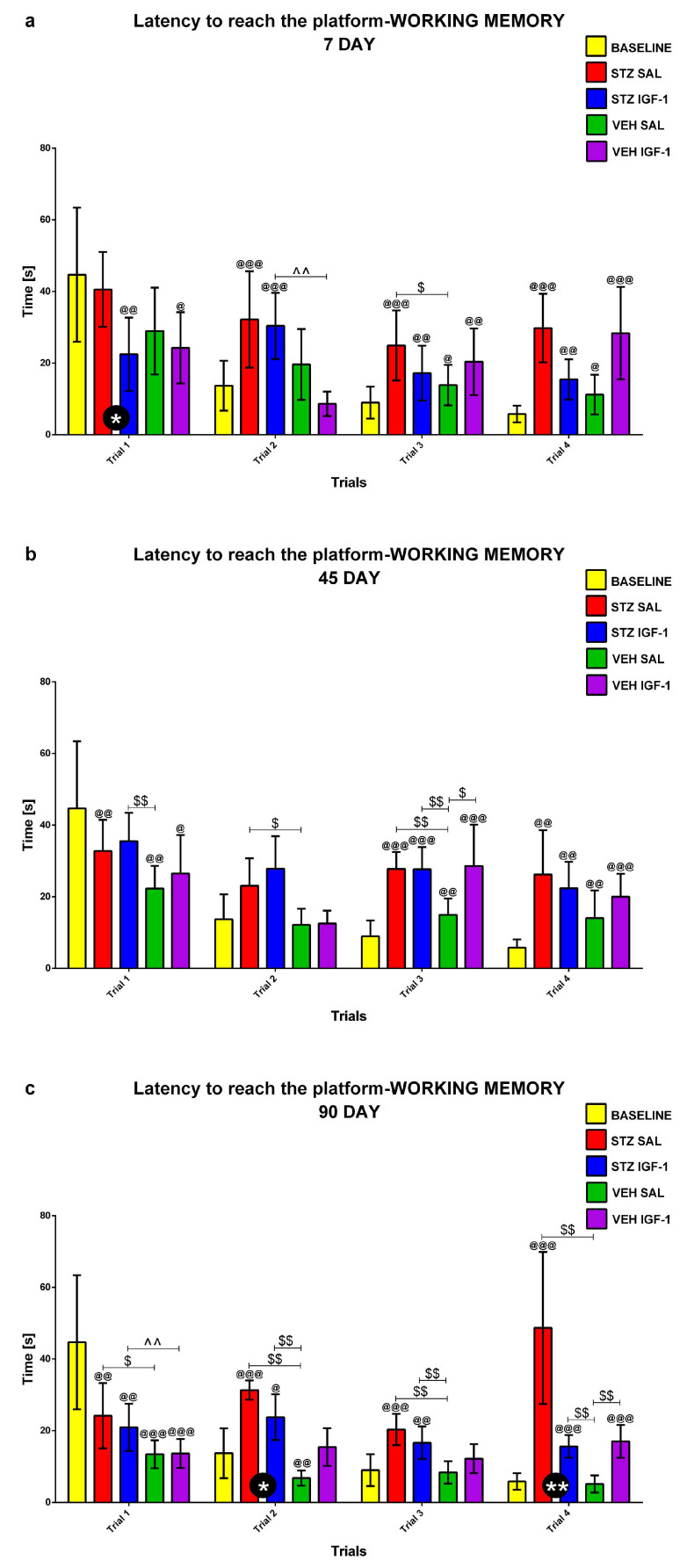
Working memory (average scores) during Trials 1–4 obtained for consecutive days 5–8 in the Morris water maze test measured as latency to reach the platform in rats before injection (BASELINE, n = 40) and starting from days 7 (**a**), 45 (**b**), and 90 (**c**) after intracerebroventricular injection of streptozotocin (STZ) and saline (SAL) (STZSAL, n = 10) or STZ and insulin-like growth factor-1 (IGF-1) (STZIGF-1, n = 10) or citrate buffer (VEH) and SAL (VEHSAL, n = 10) or VEH and IGF-1 (VEHIGF-1, n = 10). Explanations: Data are presented as mean ± SD and were analyzed using Mann–Whitney-U test; * in a circle—*p* < 0.05, ** in a circle—*p* < 0.01 difference between STZSAL and STZIGF-1 group; $ above the line between the bars -*p* < 0.05, $$ above the line between the bars—*p* < 0.01 difference vs. VEHSAL; ^^ above the line between the bars—*p* < 0.01 difference vs. VEHIGF-1; @—*p* < 0.05, @@—*p* < 0.01, @@@—*p* < 0.001 difference vs. baseline.

**Figure 6:**
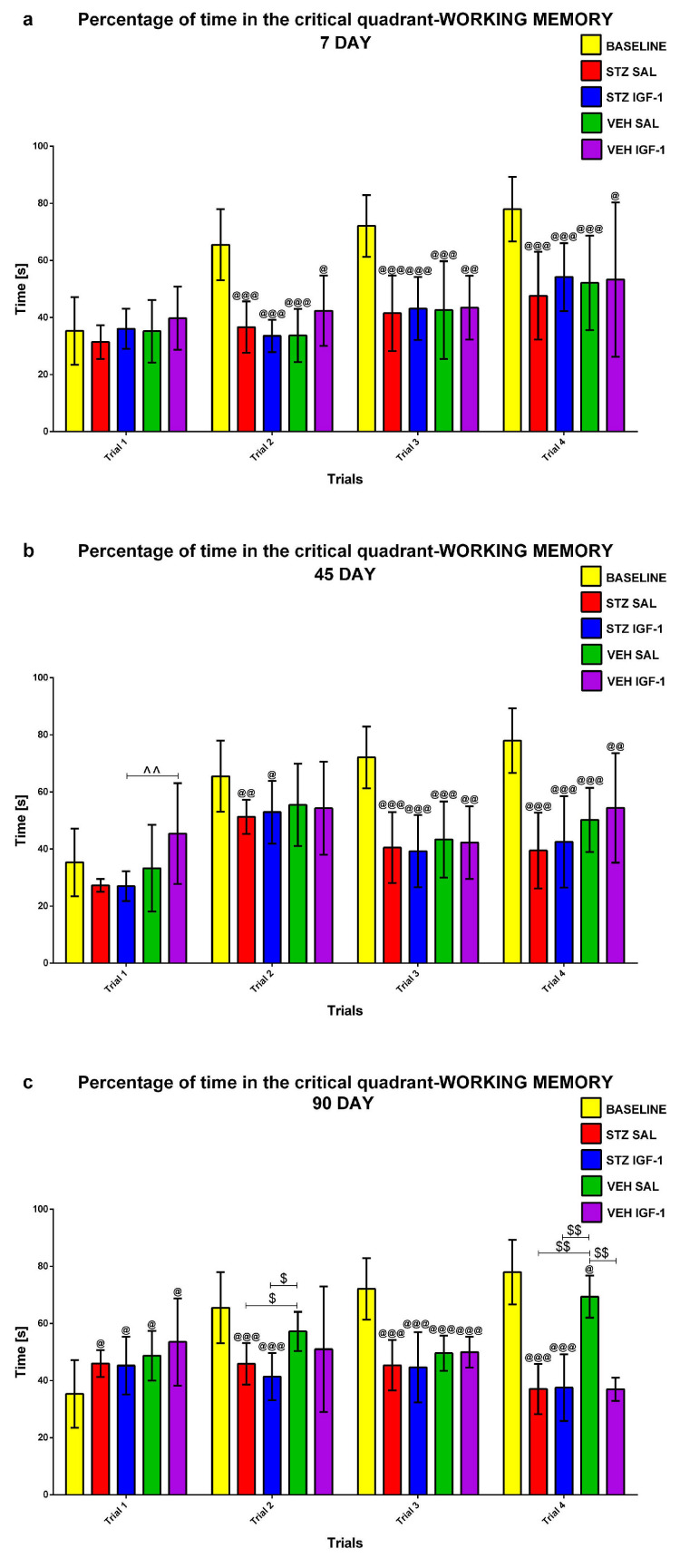
Working memory (average scores) during Trials 1–4 obtained for consecutive days 5–8 in the Morris water maze test measured as a percentage of time spent in the critical quadrant in rats before injection (BASELINE, n = 40) and 7 (**a**), 45 (**b**), and 90 (**c**) days after intracerebroventricular injection of streptozotocin (STZ) and saline (SAL) (STZSAL, n = 10) or STZ and insulin-like growth factor-1 (IGF-1) (STZIGF-1, n = 10) or citrate buffer (VEH) and SAL (VEHSAL, n = 10) or VEH and IGF-1 (VEHIGF-1, n = 10). Explanations: Data are presented as mean ± SD and were analyzed using the Mann–Whitney-U test; $ above the line between the bars—*p* < 0.05, $$ above the line between the bars—*p* < 0.01 difference vs. VEHSAL; ^^ above the line between the bars—*p* < 0.01 difference vs. VEHIGF-1; @—*p* < 0.05, @@—*p* < 0.01, @@@—*p* < 0.001 difference vs. baseline.

**Figure 7:**
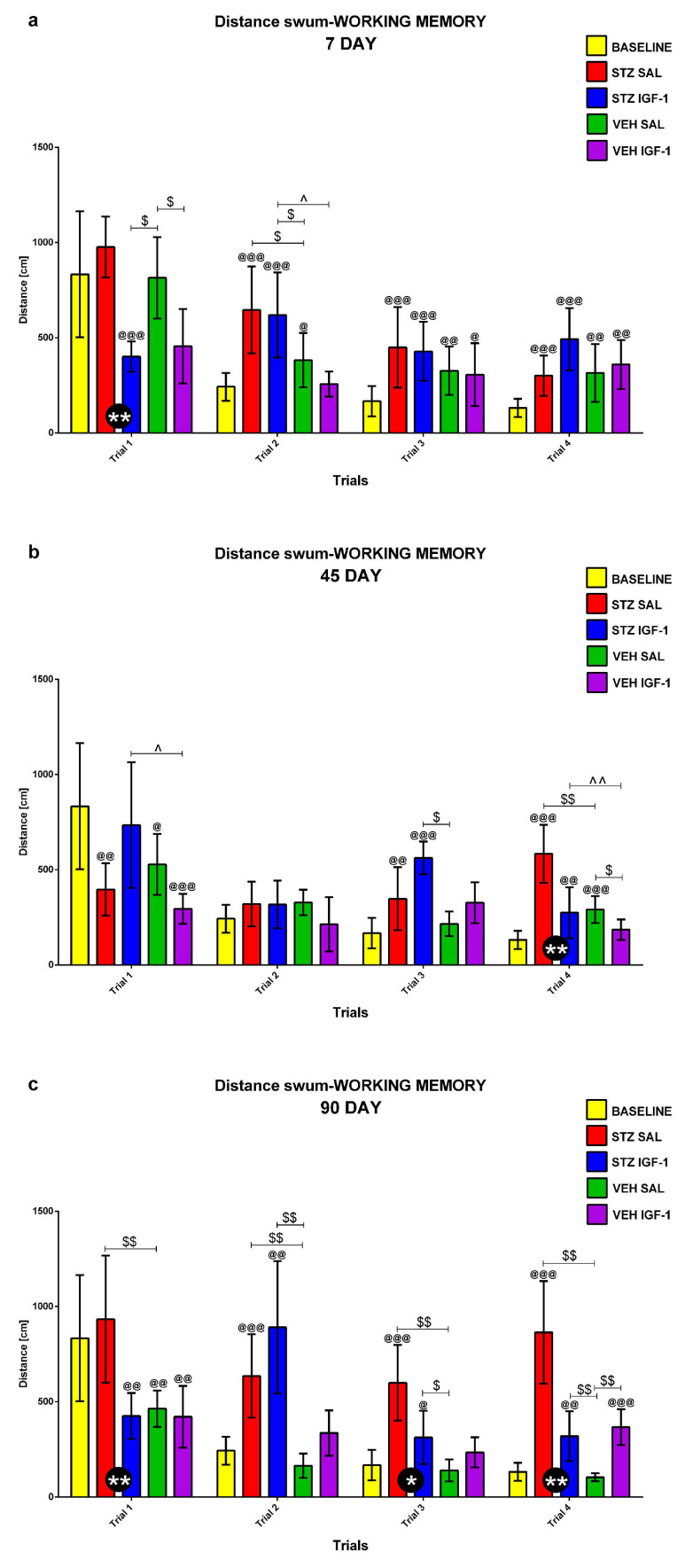
Working memory (average scores) during Trials 1–4 obtained for consecutive days 5–8 in the Morris water maze test measured as total distance swum in rats before injection (BASELINE, n = 40) and 7 (**a**), 45 (**b**), and 90 (**c**) days after intracerebroventricular injection of streptozotocin (STZ) and saline (SAL) (STZSAL, n = 10) or STZ and insulin-like growth factor-1 (IGF-1) (STZIGF-1, n = 10) or citrate buffer (VEH) and SAL (VEHSAL, n = 10) or VEH and IGF-1 (VEHIGF-1, n = 10). Explanations: Data are presented as mean ± SD and were analyzed using Mann–Whitney-U test; * in a circle—*p* < 0.05, ** in a circle—*p* < 0.01 difference between STZSAL and STZIGF-1 group; $ above the line between the bars—*p* < 0.05, $$ above the line between the bars—*p* < 0.01 difference vs. VEHSAL; ^ above the line between the bars—*p* < 0.05, ^^ above the line between the bars—*p* < 0.01 difference vs. VEHIGF-1; @—*p* < 0.05, @@—*p* < 0.01, @@@—*p* < 0.001 difference vs. baseline.

Working memory during Trials 1–4 of consecutive days 5–8 of the MWM (MWM5-MWM8) was investigated at the baseline conditions (before injection) and starting from days 7, 45, and 90 after injection. Figure 5, Figure 6 and Figure 7 present the average behavioral activity scores during Trials 1–4 across MWM5–MWM8, measured by latency to reach the platform (Figure 5), time spent in the critical quadrant (Figure 6), and total distance swum (Figure 7), comparing the STZSAL, STZIGF-1, VEHSAL, and VEHIGF-1 groups with baseline (before injection) conditions. Compared to STZSAL animals, a significantly shorter latency to reach the platform was observed during Trial 1 on day 7 (*p* < 0.05; Figure 5a), and during Trials 2 (*p* < 0.05) and 4 (*p* < 0.01) on day 90 after sAD induction in STZIGF-1 rats (Figure 5c). In addition, a significantly shorter total distance swum was observed in the STZIGF-1 group during Trial 1 on day 7 (*p* < 0.01; Figure 7a), Trial 4 on day 45 (*p* < 0.01; Figure 7b and Figure S2) and Trials 1 (*p* < 0.01), 3 (*p* < 0.05) and 4 (*p* < 0.01) at 90 days post-injection compared with STZSAL rats (Figure 7c). The only differences between the STZIGF-1 and control VEHIGF-1 groups were observed in the latency to reach the platform on day 7 post-injection during Trial 2 (Figure 5a), the percentage of time spent in the critical quadrant on day 45 during Trial 1 (Figure 6b), and the total swimming distance on day 45 during Trials 1 and 4 (Figure 7b). No significant differences were observed in the percentage of time spent in the critical quadrant (Figure 6) between the STZSAL and STZIGF-1 groups.

### 2.2. Microglia Activated Cells (CD68^+^ Cells) and Amyloid β (Aβ40–42) Deposition in the Hippocampus (CA1, CA2, CA3, DG)

**Figure 8:**
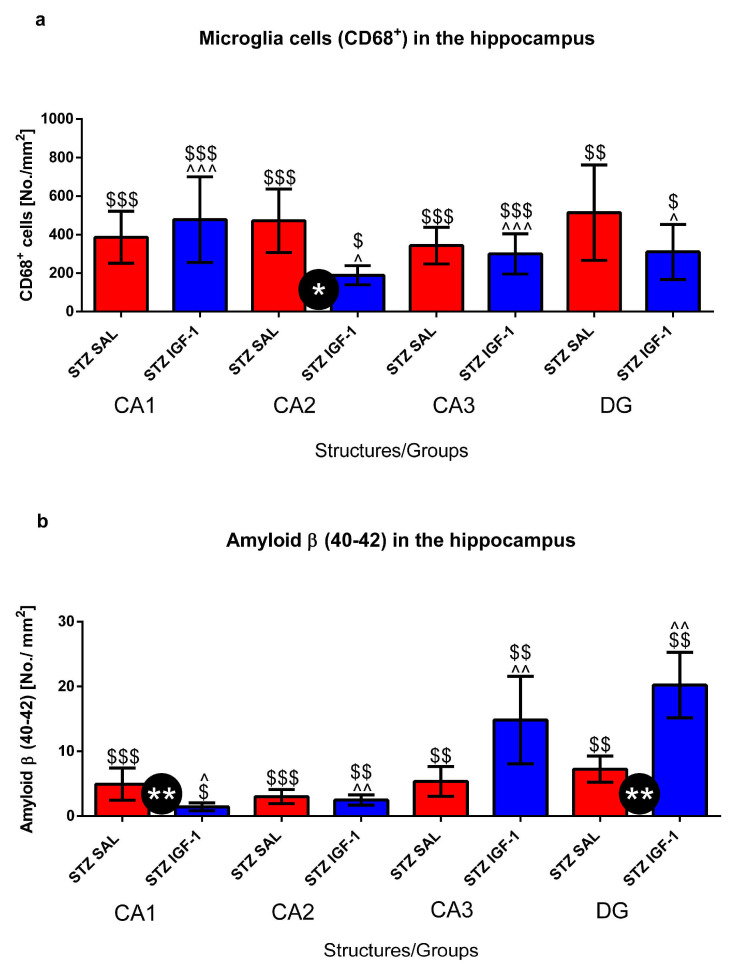
Number of activated microglia cells (CD68^+^) (**a**) and amyloid β counts (**b**) in the CA1, CA2, CA3, and DG of the hippocampus in rats at 90 days after intracerebroventricular injections of streptozotocin (STZ) and saline (SAL) (STZSAL, n = 10) or STZ and insulin-like growth factor-1 (IGF-1) (STZIGF-1, n = 10). Explanations: Data are presented as mean ± SD and were analyzed using Mann–Whitney-U test; * in a circle—*p* < 0.05, **—*p* < 0.01 differences between STZSAL and STZIGF-1; $—*p* < 0.05, $$—*p* < 0.01, $$$—*p* < 0.001 differences vs. VEHSAL; ^—*p* < 0.05, ^^—*p* < 0.01, ^^^—*p* < 0.001 differences vs. VEHIGF-1.

**Figure 9:**
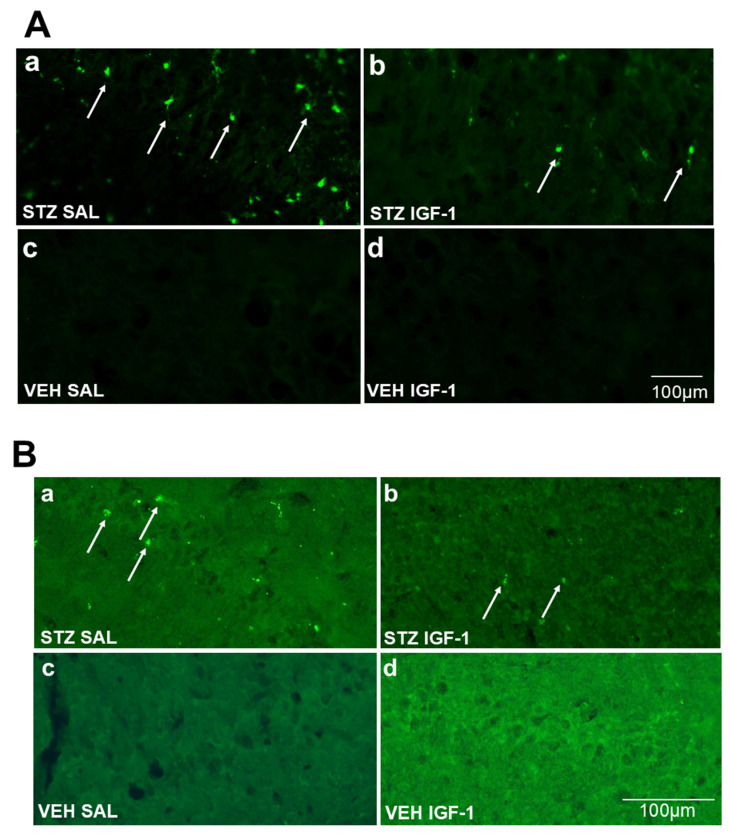
Example microscopic images showing activated microglia cells (CD68^+^ cells, indicated by arrows, magnification 10 × 20) in the CA2 area of the hippocampus (**A**) and amyloid β (Aβ40–42, indicated by arrows, magnification 10 × 10) in the CA1 area of the hippocampus (**B**) of STZSAL (**a**), STZIGF-1 (**b**), VEHSAL (**c**), and VEHIGF-1 (**d**) rats at 90 days after intracerebroventricular injections of streptozotocin (STZ) and saline (SAL) or STZ and insulin-like growth factor-1 (IGF-1) or citrate buffer (VEH) and SAL or VEH and IGF-1. Scale bar = 100 μm.

We observed significantly lower numbers of CD68^+^ microglia cells in the hippocampal CA2 (*p* < 0.05), but not in areas CA1 and CA3 (Figure 8a, Figure 9A and Figure S3A), and in the lateral preoptic area (*p* < 0.05), and supraoptic nucleus (*p* < 0.001) (Table 1) in the STZIGF-1 group, compared with STZSAL rats 90 days after AD induction. In the DG area of the hippocampus, a lower number of CD68^+^ cells was observed in STZIGF1 animals compared to STZSAL animals (Figure 8a). Furthermore, there were no activated microglia cells in all areas of the hippocampus, nucleus accumbens, prefrontal cortex, preoptic area, medial septal nucleus, caudate putamen, and hypothalamic nuclei within the VEHSAL and VEHIGF1 control groups (Table 1).

As shown in Figure 8b, Figure 9B and Figure S3B, there was a significantly (*p* < 0.01) lower number of amyloid β (Aβ) deposits in CA1, whereas there was an increased number of Aβ deposition in CA3 and DG (*p* < 0.01) in the STZIGF1 group compared with STZSAL animals. There were no significant differences in Aβ counts in the prefrontal cortex, preoptic areas, and nucleus accumbens between STZIGF-1 and STZSAL rats 90 days after STZ injection (Table 1). In addition, no Aβ deposits were found in both VEHSAL and VEHIGF-1 control groups (Table 1).

## 3. Discussion

Our results demonstrate, to the best of our knowledge for the first time, that ICVIGF-1 injection (dose of 0.5 μg administered twice on days 1 and 3, into each lateral ventricle for an accumulative dose of 2 μg,), alongside the induction of sAD via ICV administration of STZ (a cumulative dose of 3 mg/kg, administered in smaller doses twice on days 2 and 4), mitigates the deleterious effects of STZ-induced working memory impairment, and—to a lesser extent—reference memory, and reduces Aβ deposition in the brain during sAD progression. The current findings underline the significance of central IGF-1 signaling in the mechanisms and outcomes of brain insulin resistance, confirming its protective role against spatial memory deficits in sAD. Our results suggest that an effective treatment to slow or prevent early pathogenic events in sAD may involve attenuation of brain insulin resistance in AD, which highlights the therapeutic potential of future research on brain insulin resistance, including IGF-1 signaling as a novel therapeutic target for Alzheimer’s disease treatment.

Cognitive deficits are linked to insulin signaling abnormalities. Changes in the levels of various insulin signaling molecules in the forebrain of AD patients [1,33,34] have been associated with memory improvements in AD cases and those at high risk. Those changes may follow selective elevation of forebrain insulin via intranasal administration of the hormone [35,36]. Talbot et al. [33], using an ex vivo stimulation protocol with near-physiological doses of insulin and IGF-1, demonstrated that the brain in AD, particularly the hippocampal formation (CA1-CA3, DG) and, to a lesser extent, the cerebellar cortex, exhibited insulin and IGF-1 resistance. That resistance occurred in the absence of diabetes and without affecting basal neuronal glucose uptake. According to the authors, brain insulin resistance appears to be an early and common feature of AD, a phenomenon accompanied by IGF-1 resistance and closely associated with the insulin receptor substrate 1 (IRS-1) dysfunction, which is a critical element in the insulin-signaling pathway, potentially triggered by Aβ oligomers and cognitive decline.

Our findings align with previous studies supporting the protective effect of central IGF-1 recovery on spatial memory during sAD progression. We hypothesize that IGF-1 injection may prevent STZ-induced spatial memory deficits. Indeed, an improvement in reference memory, measured during MWM1-MWM3, was observed, as indicated by increased time spent in the critical quadrant, reduced total distance swum on day 45 of sAD progression, and reduced latency to reach the platform on day 90 following ICV co-injection of IGF-1 and STZ compared to STZSAL animals. The reference memory after IGF-1 administration in rats with the ICVSTZ model generally did not differ from that observed in the control VEHIGF-1 rats over three consecutive days of MWM testing. However, it did not return to the control values seen in the VEHSAL animals, especially at a later stage (90 days post-injection) of disease progression. Furthermore, there was a protective effect of ICVIGF-1 injection on working memory measured as a reduction in latency in trials 2 and 4 at the late stage of sAD progression (90 days after injection) and a reduction in total swimming distance in trials 3 and 4 at 45 and 90 days after ICVIGF-1 and ICVSTZ administration compared to the STZSAL group. However, working memory performance in the STZIGF-1 animals differed from the control values in the VEHSAL group 90 days after injection.

The results from the probe test suggest that the IGF-1-induced protective effect on reference memory performance was weak and short-term. By day 90, no significant differences were observed between the STZSAL and STZIGF-1-treated animals. In fact, control VEHIGF-1 rats showed even deterioration in memory performance compared to the VEHSAL group. Alternatively, the improvement in reference memory performance observed in STZSAL rats (without IGF-1 treatment) during the probe test at 45 and 90 days after injection could be attributed to compensatory mechanisms triggered by insulin resistance induced by long-term deleterious effects of STZ. These mechanisms may mask early cognitive dysfunction, as assessed by behavioral activity in the MWM test. Histopathological evidence of specific neurotoxic damage caused by ICVSTZ administration to axons and myelin in the fornix, anterior hippocampus, and periventricular structures essential for learning and spatial memory has been reported [37,38]. Brain insulin system dysfunction was observed 1 month after ICVSTZ administration (3 mg/kg b.w.) while an increase in total tau-protein in the brain and amyloid formation in leptomeningeal vessels was found three months after ICVSTZ injection [39], which corresponds to 90 days post-injection in our study. Additionally, cerebral amyloid angiopathy was observed 6 and 9 months after STZ-icv [40]. According to the authors, this was a continuation and progression of the amyloid pathology already observed 3 months after STZ-icv treatment. Therefore, we suggest that the lack of significant differences between the STZIGF-1 and STZSAL groups, particularly in the latency to reach the platform at 7 days post-injection, may be related to the stage of STZ-induced insulin resistance and cerebral Aβ accumulation. These factors may influence cognitive performance in different ways. After ICVSTZ administration, IGF-1 receptor gene expression was found to be reduced [41]. However, it should be considered that ICVIGF-1 treatment may have restored this important compensatory mechanism in ICVSTZ-injected rats.

A limitation of our study is the use of a rat model to study sAD in humans, and thus the translational value of animal-based results. In the present experiment, we designed three control groups as sufficient control: VEHSAL to control the entire experimental procedure (MWM, all the injections, and neuroimmune correlates), VEHIGF-1 to control the effect of IGF-1 treatment in the control rats, and intact BASELINE (naive) to control behavioral activity in the Morris water maze prior to injections in non-intact rats. However, another blank control group receiving no treatment at all might be a valuable control to minimize the influence of all potential confounding variables in the experiment and constitute a baseline for all measured parameters. Additionally, only a single dose of IGF-1 (2 μg divided into two injections) was used in our study. Future research could explore the effects of multiple doses, prolonged IGF-1 administration, or the simultaneous use of ICVIGF-1 and intranasal insulin to provide a more comprehensive understanding of IGF-1 treatment in this sAD model. However, the results of our study may support the therapeutic potential of future work on brain insulin resistance in AD and highlight the potential for combination therapies involving IGF-1 to prevent or delay dementia in humans.

Regarding the positive action of central IGF-1 in animal models, other authors reported that ICV co-administration of IGF-1 and Aβ25–35 to male rats showed its protective effects against Aβ25–35-related down-regulation of hippocampal somatostatinergic system through up-regulation of protein kinase A activity and cAMP response element-binding protein phosphorylation [42]. Additionally, Ghaffari et al. [43] demonstrated that memory and anxiogenic behavioral improvements could be attributed to the enhancement of neuronal and oligodendroglial survival and the reduction in neuroinflammation in the hippocampus in rats after ICVSTZ injection (6 mg/kg) with intranasal co-treatment of insulin (4 U/40 µL) and administration of growth factor-rich serum (1 µL/kg). According to the authors, the anti-inflammatory effect of IGF-1 could be associated with its influences on microglial and astrocyte activity. Our results also indicate that the protective effect of central IGF-1 on STZ-induced impairment of spatial memory is mediated through a reduction in neuroinflammation, as evidenced by a reduced number of activated microglial cells (CD68^+^), particularly in the CA2 area of the hippocampus and the prelimbic/hypothalamic nuclei, in the late stage of sAD progression (90 days post-injection). It is known that IGF-1 promotes an anti-inflammatory phenotype in microglia [44]. In astrocytes, IGF-1 signaling is essential for maintaining the integrity of the neurovascular unit, and its disruption may impair neurovascular coupling [45]. IGF-1 also regulates astrocytic phagocytosis and inflammation through PI3K signaling [46]. Furthermore, insulin modulates brain glucose metabolism by acting on astrocytes in cooperation with IGF-I [47]. Park et al. [48] showed that IGF-1 tempers the innate immune response within the brain and reduces the expression of inflammatory markers such as interleukin (IL)-1β, and tumor necrosis factor (TNF)-α. Nevertheless, IGF-1 can also enhance the production of anti-inflammatory cytokines, including IL-4 and IL-10.

Additionally to the central IGF-1 effect of reducing neuroinflammation, the improvement in spatial memory observed in this study can also be attributed to the protective effect of IGF-1 against Aβ deposition. Indeed, we found a reduced number of Aβ deposits, particularly in the CA1 area of the hippocampus after 90 days of sAD progression in rats ICV injected with IGF-1. However, it should be noted that we were not able to show that neuroinflammation and Aβ accumulation were significantly reduced in CA2, CA3, and DG areas of the hippocampus, as they were in CA1; yet, a trend towards reduced neuroinflammation was observed in these areas. This may suggest different roles of various hippocampal regions in insulin-resistant brain conditions. Our results are consistent with those of Carro et al. [49] who also reported that IGF-I enhances the clearance of brain Aβ by modulating the transport/production of Aβ carriers at the blood–brain interface in the choroid plexus. According to the authors, the administration of ICVIGF-1 leads to an increase in megalin protein, which mediates IGF-I-induced clearance of Aβ, thereby improving memory impairment associated with brain amyloidosis. Furthermore, our findings of improved working memory following ICVIGF-1 administration and reduced Aβ accumulation in the CA1 region of the hippocampus, are in line with other reports [50], which found that basal activation states of multiple insulin signaling molecules in CA1 pyramidal cells were highly related to episodic and working memory. Given the ability of oligomeric Aβ to inactivate IRS-1 [50,51], it may in part mediate the effects of these oligomers on cognition. In contrast to this observation, Freude et al. [52] showed that impaired IGF-1/insulin receptor substrate 2 (IRS-2) signaling prevented premature death and delayed amyloid accumulation predominantly in the hippocampus in mice with Swedish mutation of amyloid precursor protein (APP) as a model of AD and deficient for IRS-2, neuronal IGF-1 receptor or neuronal insulin receptor. The authors suggested that delayed Aβ accumulation resulted from decreased APP processing.

Besides reduced neuroinflammation and Aβ accumulation, another mechanism, including IGF-1-induced neurogenesis, may be responsible for the observed protective effects of central administration of IGF-1 on cognitive function in the ICVSTZ-induced sAD model. Insulin/IGF-1 signaling has been implicated in the regulation of adult neurogenesis in the DG [53,54]. Adult-born neurons have the ability to functionally integrate into the hippocampal circuitry and have been found to contribute to select types of hippocampal-dependent learning and memory tasks [55]. Deletion of the IGF-1 receptor gene (*Igf1r*) resulted in an almost complete loss of the DG in mice [56] and impaired learning and memory function, leading to dementia [57,58], while exogenous IGF-1 enhanced hippocampal neuronal precursor cells proliferation [59,60] and directed the generation of mature granule cells. Furthermore, IGF-1 could support synaptogenesis, in particular, in the hippocampus [5,58]. It has been reported that IGF-1 improved neuronal complexity and synaptic connectivity under insulin resistance conditions [61]. Other authors [62,63] reported acute effects of IGF-1 on excitatory synaptic transmission in the CA1 region of the hippocampus associated with attenuation of spatial learning deficits in aged rats. According to the authors, increases in AMPA receptor-mediated synaptic transmission may contribute directly to cognitive improvement or initiate long-term changes in the synthesis of proteins, such as brain-derived neurotrophic factors, that are important for neurogenesis, learning, and memory. Indeed, in our study, we observed significantly reduced Aβ accumulation in the CA1 area after IGF-1 treatment in rats with the ICVSTZ model, which also suggests the involvement of this part of the hippocampus in enhanced spatial memory observed in our study.

Furthermore, serotonin is known to promote hippocampal neurogenesis [54,64]. Many serotonergic neurons project to the hippocampal DG. It has also been reported that hippocampal neurogenesis is important for the antidepressant effects caused by antidepressants. Recently, it has been reported that agonist stimulation of serotonin type 3 (5-HT3) receptors promotes IGF-1release in the hippocampus and increases hippocampal neurogenesis via the IGF-1 signaling pathway [54], resulting in antidepressant effects [54,65], which could improve cognitive function. Clarifying whether IGF-1-induced neurogenesis and/or antidepressant effects are involved in the improvement in spatial memory observed in this study may be the subject of future research.

## 4. Materials and Methods

### 4.1. Animals

**Figure 10:**
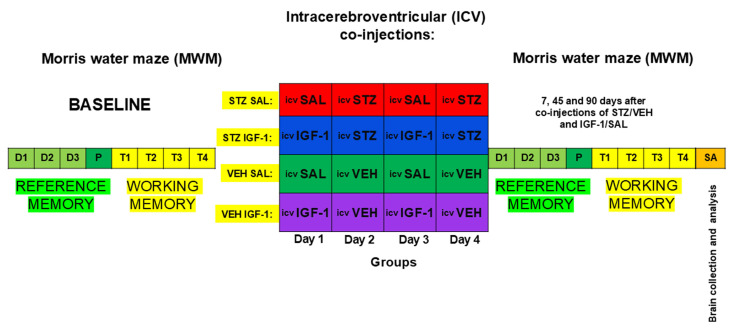
Scheme of experimental procedure and group assignments. Explanations: MWM—Morris water maze test: D1-D3 days of phase of acquisition of reference memory, P-probe test (single trial without the platform (reference memory evaluation), T1-T4 trials of working memory performance; ICVSTZ—intracerebroventricular (ICV) injections of streptozotocin (STZ) (a total dose of 3 mg/kg b.w. divided into two injections on Days 2 and 4 into each lateral ventricle: 0.75 mg/kg in 2 μL of citrate buffer per ventricle)—sAD model; ICVVEH—intracerebroventricular (ICV) injections of citrate buffer (VEH) (2 μL per ventricle); ICVIGF-1- intracerebroventricular (ICV) injections of insulin-like growth factor-1 (IGF-1) (1 µg per single administration on Days 1 and 3 into each lateral ventricle: 0.5 µg in 2 µL of saline per ventricle)—neurotrophin treatment; ICVSAL—intracerebroventricular (ICV) injections of saline (SAL) (2 μL per ventricle); S-sacrifice of animals, brain collections to future analysis (A).

All animal experiments were carried out at the University of Gdańsk, Faculty of Biology license number 0169 in accordance with Directive 2010/63/EU of the European Parliament, and under the authority of the Local Ethical Committee for the Care and Use of Laboratory Animals at University of Technology in Bydgoszcz, Poland (No. 60/2017). Male Wistar Han rats (n = 40) were obtained from a licensed breeding facility (Tri-City Central Animal Laboratory, Research and Service Centre of the Medical University of Gdansk, Poland, breeder registration number 041). Rats were allowed to adapt to the laboratory conditions for a two-week acclimatization period. Subsequently, rats were subjected to handling for two weeks in order to minimize stress during the experimental procedure. Handling lasted for about 5 min each day. During the experiment, rats were housed separately in polycarbonate cages (width 20 cm, length 40 cm, height 18 cm) on a 12 h light/dark cycle (lights on at 06:00) in an air-conditioned, constant-temperature (22 ± 2 °C) room and with water and food available ad libitum. The animals could visually observe other rats and were indirectly exposed to their cage odors. Before starting the behavioral tests under baseline conditions (before injections), the rats reached a body weight of 300 ± 20 g, corresponding to 11–12 weeks of age. The slightly higher body weight ranges of our experimental rats compared with the usual biological variability of rat body weight (10%) [66] may be due to individual differences in stress reactivity during the two-week handling procedure that preceded the body weight measurement. However, we believe that this did not affect the results of our study. After completion of the baseline behavioral measurement, the animals were randomly divided into four groups: STZSAL (n = 10), STZIGF-1 (n = 10), control VEHSAL (n = 10), control VEHIGF-1 (n = 10), according to the diagram presented in Figure 10.

### 4.2. Experimental Procedure

#### 4.2.1. Intracerebroventricular (ICV) Co-Injections of Insulin-Like Growth Factor-1 (IGF-1) and Streptozotocin (STZ)

Rats under 2.5% isoflurane anesthesia (airflow: 0.5 L per min) using an isoflurane pump (Bitmos OXY 6000, Bitmos GmbH Dűsseldorf, Germany) were prepared for surgery, at the beginning the head of the animal was immobilized using a stereotaxic apparatus (Hugo Sachs Elektronik, March-Hugstetten Germany). Next, the 3 mm steel stainless guide cannula (C311G/Spc, Bilaney, Dűsseldorf, Germany) was implanted on the surface of the skull above the positions of each lateral ventricle according to coordinates from the Paxinos and Watson [67] atlas (coordinates: AP: −1.3 mm, L: ±2 mm). The 4 mm injection needle (C311I/Spc, Bilaney), compatible with the main guide cannula, was placed in the lateral ventricles (D: +3.6 mm). Guide cannula and injection needle were a way to administer the substance. The injection needle, after drug administration, was left inside the guide cannula for the next 60 s, after which it helped to avoid a reverse flow of the solutions. The guide cannulas were permanently attached to three stainless steel skull screws using dental acrylic (Duracryl, spofa Dental a.s., Jicin, Czech Republic). After the attachment of the cannulas and stitching of the head skin, the administration of the substances was started (Day 1).

#### 4.2.2. Drug Administration

In line with our previous study using the ICVSTZ model in rats [28,29,30,31,32], in this study we used double ICV injections of STZ in a total dose of 3 mg/kg divided into two microinjections (2 × 1.5 mg/kg, dissolved in citrate buffer 0.02 M, pH 4.5) with separate microinjections into each lateral ventricle (0.75 mg/kg dissolved in 2 μL of the vehicle) on Days 2 and 4. Control rats were injected with ICV citrate buffer (VEH), following the same procedure as ICVSTZ administration. ICV administration of IGF-1 was performed according to the method and a dose of IGF-1 described previously [68,69]. A single ICV injection of IGF-1 at a dose of 1 µg, which was used in that and our studies, was sufficient to induce an improvement in behavioral activity, related to working memory, manifested as a reduction in swimming distance and latency to reach the platform in rats during trials 1–4 of the MWM test. Prior to ICV injections of IGF-1 (Biotechne, 291-G1, Minneapolis, MN, USA), the substance was diluted in saline (SAL) to obtain a dose of 0.5 µg/2 µL/ventricle. The resulting dilution allowed IGF-1 to be administered at doses of 1 µg per single administration on Days 1 and 3. Control animals received ICV saline injections using the same procedure as ICVIGF-1 administration. All co-injections of IGF-1/SAL and STZ/VEH were performed with the use of a microinfusion pump (Legato-100—Series Syringe Pump, KD SCIENTIFIC, Holliston, MA, USA) and a Hamilton syringe (capacity: 10 μL) combined with polyethylene connected to an injection cannula at a rate of 0.5 µL/min. In order to avoid a reverse flow of the solution, the injection cannula was left inside the guide for an additional 60 s. After surgery and injections, rats were moved to the worm accommodation, where they stayed until recovery.

#### 4.2.3. Behavior Associated with Reference and Working Memory in Morris Water Maze (MWM)

Morris water maze (MWM) testing was determined at baseline (before injection) conditions and at 7, 45, and 90 days post-injection. First, reference memory was measured, followed by measurements of working memory according to the methodology that we described previously [28,31,32]. The MWM training sessions with a visible, located above the water surface, platform was performed at the beginning of the behavioral testing. If the animal did not locate the platform within 1 min, it was gently directed toward the platform by the experimenter. After 5 min, a screening trial also with a visible, located above the water surface platform was performed in order to eliminate rats with sensory-motor and motivational deficits. Spatial memory testing was performed four times during the experiment, each lasting eight days, and was divided into two stages: reference memory testing with the platform remaining in constant position for all training sessions (3 days, four trials per day, and a probe test with one trial on the 4th day with platform removed, the duration of a single attempt was 2 min) and working memory testing with the platform position in the maze changed every day (4 days, 4 trials a day). The inter-trial interval (ITI) lasted 10 min. In order to minimize experimenter bias, during the exposure of the animals to the MWM test, the behavioral responses of the rats were saved using a video camera, located approximately 200 cm over the center point of the maze and connected to a video-tracking program (EthoVision XT, Noldus, Wageningen, The Netherlands). Three parameters labeled in MWM were measured with the use of a digitizing device with a video-tracking function: latency to reach the platform (in most trials) or the critical annulus (CA—virtual contour of the removed platform) in the probe test, distance swum (path length), and the percentage of time spent in the critical quadrant (CQ) of the pool (where the platform was located). Then, a trained observer who was blinded to the experimental treatment of the rats scored behavioral responses.

#### 4.2.4. Immunohistochemistry

Immunofluorescence was used for labeling activated microglia end various forms of amyloid β (oligomeric and fibrillar forms of amyloid β 42 (Aβ1–42) and non-aggregated amyloid β 40 (Aβ1–40). Since numerous studies have shown that strong expression of the lysosomal protein CD68^+^ can be used as the marker of activated microglia cells [70], we detected activated microglia (CD68^+^ cells) according to the method we described earlier [28,32]. Brains were fixed by perfusion with 4% paraformaldehyde in PBS, dehydrated in 30% sucrose, and cut in a cryostat (CM1850, Leica Wetzlar, Germany) to obtain 20 μm thick coronal sections. The sections were then blocked in buffered saline (PBS) pH 7.4 containing 5% bovine serum albumin (BSA) and 0.3% Triton X for 2 h. After triple rinsing with PBS, the sections were incubated for 48 h in a primary antibody solution: mouse monoclonal anti-CD68 (Sigma Aldrich, St. Louis, MO, USA, MAB1435, dilution 1:250) or monoclonal mouse antibody anti-amyloid β (Novus Biologicals, Centennial, CO, USA, NBP2-13075, 1:300) in PBS containing 0.3% Triton X and 2% BSA. After three times rinsing, the sections were incubated for 2 h in a secondary antibody solution (goat anti-mouse, Abcam, Cambridge, United Kindom, Alexa Fluor 488, ab150113, 1:500 in PBS) for both CD68^+^ microglia and Aβ. Next, the sections were rinsed three times, placed on gelatinized slides, and overslipped with the use of Fluoromount-G solution with DAPI (Thermo Fisher Scientific, Waltham, MA, USA 00-4959-52). The slides containing CD68^+^ microglia were photographed with a Zeiss fluorescent microscope (Axio Scope A1, Oberkochen, Germany) compatible with the Axio Vision software 4.8.2. The prepared slides of Aβ were photographed using a fluorescence microscope (DM1000 LED, Leica, Wetzlar, Germany) compatible with the LAS X Multi Channel program.

#### 4.2.5. Quantification of Labeled Cells

Images were taken on a blue channel (DAPI) and a green channel (CD68 or amyloid β), but when the two channels were combined into one image, it was found that the DAPI channel generated high background fluorescence. Therefore, quantification of CD68^+^ cells and amyloid β aggregates was performed on an image containing only the main green channel in order to accurately identify the labeled objects.

Counting of microglia cells and beta-amyloid deposits was performed by an observer blinded to the experimental group allocation. CD68-positive (CD68^+^) cells and beta-amyloid (Aβ) positive aggregates were counted in both hemispheres of each tested structure. The borders of the brain structures were determined on the basis of the Paxinos and Watson atlas [67]. Active microglia cells (CD68^+^) were counted in a calibrated frame of 0.01 mm^2^, and Aβ aggregates were counted in a calibrated frame of 0.1 mm^2^ area imposed in the software. The density of CD68^+^ cells or Aβ aggregates was computed (number per 1 mm^2^) in each tested area. When possible, the value of three separate counts including both brain hemispheres of an individual brain region for each animal (three sections) was averaged and used as a single data point.

#### 4.2.6. Slaughtering Animals and Collection of Tissues

Rats were sacrificed with a lethal dose (2 mL/kg i.p.) of Morbital (Biowet, Puławy, Poland) under 2.5% isoflurane anesthesia (airflow: 0.5 L per min). Then, perfusion (throughout the left ventricle of the heart) using PBS (approx. 200 mL/per rat) and 4% paraformaldehyde solution (approx. 200 mL/per rat) was carried out. After the perfusion was completed, the brain was collected and immediately placed in a 4% paraformaldehyde solution, where it stayed for about 4 days. The fixed brain was dehydrated with 30% sucrose solution and then placed in a low-temperature freezer (−70 °C).

#### 4.2.7. Data Analysis

For a statistical analysis of the results, Statistica 13.1.336.0 software (Statsoft, Tulsa, OK, USA), run on a PC-compatible computer, was used. The data are presented as the mean ± SD. The normality of the distribution of the variables was checked with the Kołmogorow-Smirnov test and the homogeneity of the variances with a Levene test. As the outcome of the Kołmogorow-Smirnov test indicated that assumptions of parametric analysis were not fulfilled, we used it for further statistical evaluation of differences in behavioral activity and brain correlates, non-parametric statistical tests. Data were analyzed using the Kruskal–Wallis ANOVA by ranks for multiple comparisons (time: baseline, 7, 45, and 90 days post-injection; and treatment: STZ, IGF-1, VEH, SAL) and the Mann–Whitney U test for comparisons between two groups of rats. A P value lower than 0.05 was considered statistically significant.

## 5. Conclusions

The results show that double ICV injections of IGF-1 at a cumulative dose of 2 μg attenuate the deleterious effects of ICVSTZ administration on working and reference memory deficits in a rat model of sAD. The protective effect of central IGF-1 was mediated by a reduction in STZ-induced neuroinflammation, including a decrease in the number of activated microglia cells (CD68^+^) in the hippocampus and supra/preoptic hypothalamic area, and reduced brain Aβ deposition. Our data indicate that improving central insulin signaling, which targets IGF-1, may enhance cognitive function impaired during sAD progression. This suggests the potential role of IGF-1 as a novel therapeutic target, also in combination with insulin, in therapeutic strategies to repair impaired glucose metabolism in the treatment of dementia in sporadic Alzheimer’s disease and type 2 diabetes.

## Figures and Tables

**Table 1:** Number of activated microglia cells (CD68^+^ cells) (a,b) and β-amyloid counts (c,d) in brain structures of rats at 90 days after intracerebroventricular co-injection of streptozotocin (STZ) and saline (SAL) (STZSAL, n = 10) or STZ and insulin-like growth factor-1 (IGF-1) (STZIGF-1, n = 10) or citrate buffer (VEH) and SAL (VEHSAL, n = 10) or VEH and IGF-1 (VEH IGF-1, n = 10).

**Microglia Cells (CD68^+^) No./mm^2^**					
a	**Structures/Groups**	**STZ SAL**	**STZ IGF-1**	**VEH SAL**	**VEH IGF-1**
	Prefrontal cortex	0 ± 0	0 ± 0	0 ± 0	0 ± 0
	Corpus calosum	560 ± 151.66	475 ± 103.51	300 ± 115	371.43 ± 131.39
	Caudate putamen	242.86 ± 85.91 ^$^	287.5 ± 105.01 ^$$$^^^	0 ± 0	22.22 ± 11.03
	Medial septal nucleus	142.86 ± 61.00	0 ± 0	0 ± 0	0 ± 0
	Lateral preoptic area	57.14 ± 26.73 ^$^	0 ± 0 *	0 ± 0	0 ± 0
	Medial preoptic nucleus	0 ± 0	0 ± 0	0 ± 0	0 ± 0
	Supraoptic nucleus	550 ± 207.36 ^$$^	0 ± 0 ***	0 ± 0	37.5 ± 12.93
	Arcuate hypothalamic nucleus	0 ± 0	0 ± 0	0 ± 0	0 ± 0
	Dorsomedial hypothalamic nucleus	42.86 ± 13.37	0 ± 0	0 ± 0	0 ± 0
	Lateral hypothalamus	0 ± 0	0 ± 0	0 ± 0	0 ± 0
	Paraventricular hypothalamic nucleus	114.29 ± 36.60	0 ± 0	0 ± 0	0 ± 0
	Ventromedial hypothalamic nucleus	0 ± 0	60 ± 29.44	0 ± 0	0 ± 0
	Nucleus accumbens-Shell	71.42 ± 37.40	0 ± 0	0 ± 0	0 ± 0
	Nucleus accumbens-Core	57.14 ± 19.67	0 ± 0	0 ± 0	0 ± 0
b	Structures/Groups	VEH SAL		VEH IGF-1	
	Hippocampus CA1	0 ± 0		0 ± 0	
	Hippocampus CA2	0 ± 0		0 ± 0	
	Hippocampus CA3	0 ± 0		0 ± 0	
	Dentate gyrus	0 ± 0		0 ± 0	
**Amyloid** **β** **(40–42)/No./mm^2^**					
c	**Structures/Groups**	**STZ SAL**	**STZ IGF-1**	**VEH SAL**	**VEH IGF-1**
	Prefrontal cortex	3 ± 1.06 ^$^	03.06 ± 1.72	0 ± 0	0 ± 0
	Lateral preoptic area	3 ± 1.13 ^$$^	2.65 ± 0.97 ^$$^^^	0 ± 0	0 ± 0
	Medial preoptic nucleus	5.38 ± 2.36 ^$$^	1.99 ± 0.63 ^$$^^^	0 ± 0	0 ± 0
	Nucleus accumbens-Shell	2.35 ± 1.5 ^$^	3.27 ± 1.34 ^$$^^^^	0 ± 0	0 ± 0
	Nucleus accumbens-Core	2.75 ± 0.86 ^$$$^	02.16 ± 0.96 ^$$^^^^	0 ± 0	0 ± 0
d	**Structures/Groups**	**VEH SAL**		**VEH IGF-1**	
	Hippocampus CA1	0 ± 0		0 ± 0	
	Hippocampus CA2	0 ± 0		0 ± 0	
	Hippocampus CA3	0 ± 0		0 ± 0	
	Dentate gyrus	0 ± 0		0 ± 0	

Explanations: Data are presented as mean ± SD and were analyzed using Mann–Whitney-U test; *-*p* < 0.05, ***-*p* < 0.001 differences between STZSAL and STZIGF-1 group; $-*p* < 0.05, $$-*p* < 0.01, $$$-*p* < 0.001 differences vs. VEHSAL group; ^^-*p* < 0.01, ^^^-*p* < 0.001 differences vs. VEHIGF-1 group.

## Data Availability

Data are contained within the article and Supplementary Materials.

## Supplementary Materials

The following supporting information can be downloaded at https://www.mdpi.com/article/10.3390/ph18040527/s1, Figure S1. Visualization of spatial memory performance in Morris water maze (MWM) test illustrating the time spent in each quadrant of the pool (a. heat maps) and distance swum (b. path shapes) of representative rats of all groups at baseline and 8 days after ICV co-injections of streptozotocin (STZ) and saline (STZSAL), STZ and insulin-like growth factor-1 (IGF-1) (STZIGF-1), citrate buffer and saline (VEHSAL), citrate buffer and IGF-1, during the first trial on the second day of reference memory testing (MWM2). During the trial, the platform was located in the center of the lower left quadrant. In heat maps (Figure S1a), the longer time stay of an individual in a given location was marked with a warmer color (red part). Figure S2. Visualization of spatial memory performance in Morris water maze (MWM) test illustrating the time spent in each quadrant of the pool (a. heat maps) and distance swum (b. path shapes) of representative rats of all groups on the baseline and 49–52 days after ICV co-injection of streptozotocin (STZ) and saline (STZSAL), STZ and insulin-like growth factor-1 (IGF-1) (STZIGF-1), citrate buffer and saline (VEHSAL), citrate buffer and IGF-1, during four trails of 5–8 consecutive days of working memory testing. During the trials, the platform was placed in one of the centers of each quadrant. In heat maps (Figure S2a), the longer time stay of an individual in a given location was marked with a warmer color (red part). Figure S3. Original microscopy images showing CD68^+^ microglia cells in the CA2 area of the hippocampus (5Aa (STZSAL), 5Ab (STZIGF-1), 5Ac (VEHSAL), 5Ad (VEHIGF-1), fluorescence microscope Zeiss Axio Scope A1, Oberkochen, Germany, compatible with the Axio Vision software 4.8.2.) and amyloid β (Aβ40–42) in the CA1 area of the hippocampus (5Ba (STZSAL), 5Bb (STZIGF-1), 5Bc (VEHSAL), 5Bd (VEHIGF-1), fluorescence microscope DM1000 LED, Leica, Wetzlar, Germany, compatible with the LAS X Multi Channel program) of rats at 90 days after intracerebroventricular injections of streptozotocin (STZ) and saline (SAL) or STZ and insulin-like growth factor-1 (IGF-1) or citrate buffer (VEH) and SAL or VEH and IGF-1. Figure S3 is also available at the link https://drive.google.com/drive/folders/10BoB2fpfAjr2IH2lgMT7GZnMeVjlPkcE?usp=sharing (accessed on 10 March 2025).

## Abbreviations

The following abbreviations are used in this manuscript:

IGF-1	Insulin-like growth factor-1
SAL	Saline
Aβ	Amyloid βeta
STZ	Streptozotocin
VEH	Citrate buffer
AD	Alzheimer’s disease
sAD	Sporadic Alzheimer’s disease
CD68^+^ cells	activated microglia cells
ICV	Intracerebroventricular
MWM	Morris Water Maze test
CA1 CA2	Cornus ammonis 1 area of the hippocampus Cornus ammonis 2 area of the hippocampus
CA3	Cornus ammonis 3 area of the hippocampus
DG	Dentate gyrus area of the hippocampus
IL-10	Interleukin-10
IL-4	Interleukin-4
TNF-α	Tumor necrosis factor-α
IL-1β	Interleukin-1β
APP	Amyloid precursor protein
BDNF	Brain-derived neurotrophic factor
IRS-1	Insulin receptor substrate 1
IRS-2	Insulin receptor substrate 2
cAMP	Cyclic adenosine monophosphate
PI3K PBS	Phosphoinositide 3-kinase-protein kinase B Phosphate-buffered saline

## References

[1] Miao J., Zhang Y., Su C., Zheng Q., Guo J. (2024). Insulin-like growth factor signaling in Alzheimer’s disease: Pathophysiology and therapeutic strategies. Mol. Neurobiol..

[2] Qiao X., Yan J., Zang Z., Xi L., Zhu W., Zhang E., Wu L. (2024). Association between IGF-1 levels and MDD: A case-control and meta-analysis. Front. Psychiatry.

[3] Gray S.C., Kinghorn K.J., Woodling N.S. (2020). Shifting equilibriums in Alzheimer’s disease: The complex roles of microglia in neuroinflammation, neuronal survival and neurogenesis. Neural Regen. Res..

[4] El-Hakim Y., Mani K.K., Pickle K.A., Akbari Z., Samiya N., Pham C., Salas G., Pilla R., Sohrabji F. (2024). Peripheral, but not central, IGF-1 treatment attenuates stroke-induced cognitive impairment in middle-aged female Sprague Dawley rats: The gut as a therapeutic target. Brain Behav. Immun..

[5] Zhao F., Siu J.J., Huang W., Askwith C., Cao L. (2019). Insulin modulates excitatory synaptic transmission and synaptic plasticity in the mouse hippocampus. Neuroscience.

[6] Takeishi J., Tatewaki Y., Nakase T., Takano Y., Tomita N., Yamamoto S., Mutoh T., Taki Y. (2021). Alzheimer’s disease and type 2 diabetes mellitus: The use of MCT oil and a ketogenic diet. Int. J. Mol. Sci..

[7] Zappa Villar M.F., López Hanotte J., Crespo R., Pardo J., Reggiani P.C. (2021). Insulin-like growth factor 1 gene transfer for sporadic Alzheimer’s disease: New evidence for trophic factor mediated hippocampal neuronal and synaptic recovery-based behavior improvement. Hippocampus.

[8] Chami B., Steel A.J., De La Monte S.M., Sutherland G.T. (2016). The rise and fall of insulin signaling in Alzheimer’s disease. Metab. Brain Dis..

[9] Dineley K.T., Jahrling J.B., Denner L. (2014). Insulin resistance in Alzheimer’s disease. Neurobiol. Dis..

[10] Lewitt M.S., Boyd G.W. (2019). The role of insulin-like growth factors and insulin-like growth factor-binding proteins in the nervous system. Biochem. Insights.

[11] Fluca A.L., Pani B., Janjusevic M., Zwas D.R., Abraham Y., Calligaris M., Beltrami A.P., Campos Corgosinho F., Marketou M., D’Errico S. (2024). Unraveling the relationship among insulin resistance, IGF-1, and amyloid-beta 1-40: Is the definition of type 3 diabetes applicable in the cardiovascular field?. Life Sci..

[12] Bi A., An W., Wang C., Hua Y., Fang F., Dong X., Chen R., Zhang Z., Luo L. (2020). SCR-1693 inhibits tau phosphorylation and improves insulin resistance associated cognitive deficits. Neuropharmacology.

[13] Mishra S.K., Singh S., Shukla S., Shukla R. (2018). Intracerebroventricular streptozotocin impairs adult neurogenesis and cognitive functions via regulating neuroinflammation and insulin signaling in adult rats. Neurochem. Int..

[14] Ma N., Liang Y., Yue L., Liu P., Xu Y., Zhu C. (2022). The identities of insulin signaling pathway are affected by overexpression of Tau and its phosphorylation form. Front. Aging Neurosci..

[15] Hamzé R., Delangre E., Tolu S., Moreau M., Janel N., Bailbé D., Movassat J. (2022). Type 2 diabetes mellitus and Alzheimer’s disease: Shared molecular mechanisms and potential common therapeutic targets. Int. J. Mol. Sci..

[16] Bhalla S., Mehan S., Khan A., Rehman M.U. (2022). Protective role of IGF-1 and GLP-1 signaling activation in neurological dysfunctions. Neurosci. Biobehav. Rev..

[17] Huffman D.M., Farias Quipildor G., Mao K., Zhang X., Wan J., Apontes P., Cohen P., Barzilai N. (2016). Central insulin-like growth factor-1 (IGF-1) restores whole-body insulin action in a model of age-related insulin resistance and IGF-1 decline. Aging Cell.

[18] Farias Quipildor G.E., Mao K., Hu Z., Novaj A., Cui M.H., Gulinello M., Branch C.A., Gubbi S., Patel K., Moellering D.R. (2019). Central IGF-1 protects against features of cognitive and sensorimotor decline with aging in male mice. Geroscience.

[19] Gubbi S., Quipildor G.F., Barzilai N., Huffman D.M., Milman S. (2018). 40 years of IGF1: IGF1: The Jekyll and Hyde of the aging brain. J. Mol. Endocrinol..

[20] Ashpole N.M., Herron J.C., Mitschelen M.C., Farley J.A., Logan S., Yan H., Ungvari Z., Hodges E.L., Csiszar A., Ikeno Y. (2016). IGF-1 regulates vertebral bone aging through sex-specific and time-dependent mechanisms. J. Bone Miner. Res..

[21] Hossain M.A., Adithan A., Alam M.J., Kopalli S.R., Kim B., Kang C.W., Hwang K.C., Kim J.H. (2021). IGF-1 facilitates cartilage reconstruction by regulating PI3K/AKT, MAPK, and NF-kB signaling in rabbit osteoarthritis. J. Inflamm. Res..

[22] Herrera M.L., Camparini L.G., Oliveros A.L., Bellini M.J., Herenu C.B. (2024). Potentialities of IGF-1 for regulating oxidative stress in neuroinflammation and neurodegeneration: Theoretical review. Explor. Neuroprot. Ther..

[23] Salkovic-Petrisic M., Knezovic A., Hoyer S., Riederer P. (2013). What have we learned from the streptozotocin-induced animal model of sporadic Alzheimer’s disease, about the therapeutic strategies in Alzheimer’s research. J. Neural. Transm..

[24] Hoyer S., Lannert H. (2007). Long-term abnormalities in brain glucose/energy metabolism after inhibition of the neuronal insulin receptor: Implication of tau-protein. J. Neural. Transm. Suppl..

[25] Agrawal R., Tyagi E., Shukla R., Nath C. (2011). Insulin receptor signaling in rat hippocampus: A study in STZ (ICV) induced memory deficit model. Eur. Neuropsychopharmacol..

[26] Rai S., Kamat P.K., Nath C., Shukla R. (2014). Glial activation and post-synaptic neurotoxicity: The key events in streptozotocin (ICV) induced memory impairment in rats. Pharmacol. Biochem. Behav..

[27] Fuster-Matanzo A., Llorens-Martín M., Hernández F., Avila J. (2013). Role of neuroinflammation in adult neurogenesis and Alzheimer disease: Therapeutic approaches. Mediat. Inflamm..

[28] Majkutewicz I., Kurowska E., Podlacha M., Myślińska D., Grembecka B., Ruciński J., Plucińska K., Jerzemowska G., Wrona D. (2016). Dimethyl fumarate attenuates intracerebroventricular streptozotocin-induced spatial memory impairment and hippocampal neurodegeneration in rats. Behav. Brain Res..

[29] Majkutewicz I., Kurowska E., Podlacha M., Myślińska D., Grembecka B., Ruciński J., Pierzynowska K., Wrona D. (2018). Age-dependent effects of dimethyl fumarate on cognitive and neuropathological features in the streptozotocin-induced rat model of Alzheimer’s disease. Brain Res..

[30] Kurowska-Rucińska E., Ruciński J., Myślińska D., Grembecka B., Wrona D., Majkutewicz I. (2022). Dimethyl fumarate alleviates adult neurogenesis disruption in hippocampus and olfactory bulb and spatial cognitive deficits induced by intracerebroventricular streptozotocin injection in young and aged rats. Int. J. Mol. Sci..

[31] Wrona D., Majkutewicz I., Świątek G., Dunacka J., Grembecka B., Glac W. (2022). Dimethyl fumarate as the peripheral blood inflammatory mediators inhibitor in prevention of streptozotocin-induced neuroinflammation in aged rats. J. Inflamm. Res..

[32] Dunacka J., Świątek G., Wrona D. (2024). High behavioral reactivity to novelty as a susceptibility factor for memory and anxiety disorders in streptozotocin-induced neuroinflammation as a rat model of Alzheimer’s disease. Int. J. Mol. Sci..

[33] Talbot K., Wang H.Y., Kazi H., Han L.Y., Bakshi K.P., Stucky A., Fuino R.L., Kawaguchi K.R., Samoyedny A.J., Wilson R.S. (2012). Demonstrated brain insulin resistance in Alzheimer’s disease patients is associated with IGF-1 resistance, IRS-1 dysregulation, and cognitive decline. J. Clin. Investig..

[34] Moloney A.M., Griffin R.J., Timmons S., O’Connor R., Ravid R., O’Neill C. (2010). Defects in IGF-1 receptor, insulin receptor and IRS-1/2 in Alzheimer’s disease indicate possible resistance to IGF-1 and insulin signaling. Neurobiol. Aging.

[35] Reger M.A., Watson G.S., Green P.S., Baker L.D., Cholerton B., Fishel M.A., Plymate S.R., Cherrier M.M., Schellenberg G.D., Frey W.H. (2008). Intranasal insulin administration dose-dependently modulates verbal memory and plasma amyloid-β in memory-impaired older adults. J. Alzheimers Dis..

[36] Craft S., Baker L.D., Montine T.J., Minoshima S., Watson G.S., Claxton A., Arbuckle M., Callaghan M., Tsai E., Plymate S.R. (2012). Intranasal insulin therapy for Alzheimer disease and amnestic mild cognitive impairment, a pilot clinical trial. Arch. Neurol..

[37] Shoham S., Bejar C., Kovalev E., Weinstock M. (2023). Intracerebroventricular injection of streptozotocin causes neurotoxicity to myelin that contributes to spatial memory deficits in rats. Exp. Neurol..

[38] Grűnblatt E., Salkovic-Petrisic M., Osmanovic J., Riederer P., Hoyer S. (2007). Brain insulin system dysfunction in streptozotocin intracerebroventriculary treated rats generates hyperphosphorylated tau protein. J. Neurochem..

[39] Salkovic-Petrisic M., Tribl F., Schmidt M., Hoyer S., Riederer P. (2006). Alzheimer-like changes in protein kinase B and glycogen synthase kinase-3 in rat frontal cortex and hippocampus after damage to the insulin signaling pathway. J. Neurochem..

[40] Salkovic-Petrisic M., Osmanovic-Barilar J., Bruckner M.K., Hoyer S., Arendt T., Riederer P. (2011). Cerebral amyloid angiopathy in streptozotocin rat model of sporadic Alzhimer’s disease: A long-term follow up study. J. Neural. Transm..

[41] Grűnblatt E., Hoyer S., Riederer P. (2004). Gene expression profile in streptozotocin rat model for sporadic Alzheimer’s disease. J. Neural Transm..

[42] Aguado-Llera D., Canelles S., Frago L.M., Chowen J.A., Argente J., Arilla E., Barrios V. (2018). The protective effects of IGF-I against β-amyloid-related downregulation of hippocampal somatostatinergic system involve activation of Akt and protein kinase A. Neuroscience.

[43] Ghaffari M.K., Rafati A., Karbalaei N., Haghani M., Nemati M., Sefati N., Namavar M.R. (2024). The effect of intra-nasal co-treatment with insulin and growth factor-rich serum on behavioral defects, hippocampal oxidative-nitrosative stress, and histological changes induced by icv-STZ in a rat model. Naunyn-Schmiedeberg’s Arch. Pharmacol..

[44] Sun Z., Wu K., Gu L., Huang L., Zhuge Q., Yang S., Wang Z. (2020). IGF-1R stimulation alters microglial polarization via TLR4/NF-κB pathway after cerebral hemorrhage in mice. Brain Res. Bull..

[45] Toth L., Czigler A., Hegedus E., Komaromy H., Amrein K., Czeiter E., Yabluchanskiy A., Koller A., Orsi G., Perlaki G. (2022). Age-related decline in circulating IGF-1 associates with impaired neurovascular coupling responses in older adults. GeroScience.

[46] Pinto-Benito D., Paradela-Leal C., Ganchala D., de Castro-Molina P., Arevalo M.A. (2022). IGF-1 regulates astrocytic phagocytosis and inflammation through the p110α isoform of PI3K in a sex-specific manner. Glia.

[47] Fernandez A.M., Hernandez-Garzón E., Perez-Domper P., Perez-Alvarez A., Mederos S., Matsui T., Santi A., Trueba-Saiz A., García-Guerra L., Pose-Utrilla J. (2017). Insulin regulates astrocytic glucose handling through cooperation with IGF-I. Diabetes.

[48] Park S.E., Dantzer R., Kelley K.W., McCusker R.H. (2011). Central administration of insulin-like growth factor-I decreases depressive-like behavior and brain cytokine expression in mice. J. Neuroinflammation.

[49] Carro E., Spuch C., Trejo J.L., Antequera D., Torres-Aleman I. (2005). Choroid plexus megalin is involved in neuroprotection by serum insulin-like growth factor I. J. Neurosci..

[50] Ma Q.L., Yang F., Rosario E.R., Ubeda O.J., Beech W., Gant D.J., Chen P.P., Hudspeth B., Chen C., Zhao Y. (2009). β-amyloid oligomers induce phosphorylation of tau and inactivation of insulin receptor substrate via c-Jun N-terminal kinase signaling: Suppression by omega-3 fatty acids and curcumin. J. Neurosci..

[51] Bomfim T.R., Forny-Germano L., Sathler L.B., Brito-Moreira J., Houzel J.C., Decker H., Silverman M.A., Kazi H., Melo H.M., McClean P.L. (2012). An anti-diabetes agent protects the mouse brain from defective insulin signaling caused by Alzheimer’s disease–associated Aβ oligomers. J. Clin. Investig..

[52] Freude S., Hettich M.M., Schumann C., Stöhr O., Koch L., Köhler C., Udelhoven M., Leeser U., Müller M., Kubota N. (2009). Neuronal IGF-1 resistance reduces Abeta accumulation and protects against premature death in a model of Alzheimer’s disease. FASEB J..

[53] Ziegler A.N., Levison S.W., Wood T.L. (2015). Insulin and IGF-1 receptor signaling in neural-stem-cell homeostasis. Nat. Rev. Endocrinol..

[54] Kondo M. (2023). Molecular mechanisms of exercise-induced hippocampal neurogenesis and antidepressant effects. JMA J..

[55] Mir S., Cai W., Carlson S.W., Saatman K.E., Andres D.A. (2017). IGF-1 mediated neurogenesis involves a Novel RIT1/Akt/Sox2 Cascade. Sci. Rep..

[56] Liu W., Ye P., O’Kusky J.R., D’Ercole A.J. (2009). Type 1 insulin-like growth factor receptor signaling is essential for the development of the hippocampal formation and dentate gyrus. J. Neurosci. Res..

[57] Soto M., Cai W., Konishi M., Kahn C.R. (2019). Insulin signaling in the hippocampus and amygdala regulates metabolism and neurobehavior. Proc. Natl. Acad. Sci. USA.

[58] Hayes C.A., Wilson D., De Leon M.A., Jolayemi Mustapha M., Morale S., Odden M.C., Ashpole N.M. (2025). Insulin-like growth factor-1 and cognitive health: Exploring cellular, preclinical, and clinical dimensions. Front. Neuroendocrinol..

[59] Blume G.R., Royes L.F.F. (2024). Peripheral to brain and hippocampus crosstalk induced by exercise mediates cognitive and structural hippocampal adaptations. Life Sci..

[60] Kim Y.K., Jo D., Arjunan A., Ryu Y., Lim Y.H., Choi S.Y., Kim H.K., Song J. (2024). Identification of IGF-1 effects on white adipose tissue and hippocampus in Alzheimer’s disease mice via transcriptomic and cellular analysis. Int. J. Mol. Sci..

[61] Jo D., Choi S.Y., Ahn S.Y., Song J. (2025). IGF1 enhances memory function in obese mice and stabilizes the neural structure under insulin resistance via AKT-GSK3β-BDNF signaling. Biomed. Pharmacother..

[62] Akbari S., Haghani M., Ghobadi M., Hooshmandi E., Haghighi A.B., Salehi M.S., Pandamooz S., Azarpira N., Afshari A., Zabihi S. (2023). Combination therapy with platelet-rich plasma and epidermal neural crest stem cells increases treatment efficacy in vascular dementia. Stem Cells Int..

[63] Kim S.H., Kim C.H. (2024). Neuronal IGF-1 overexpression restores hippocampal newborn cell survival and recent CFC memory consolidation in Ca_v_1.3 knock-out mice. Brain Res..

[64] Azargoonjahromi A. (2024). Serotonin enhances neurogenesis biomarkers, hippocampal volumes, and cognitive functions in Alzheimer’s disease. Mol. Brain.

[65] Kondo M., Koyama Y., Nakamura Y., Shimada S. (2018). A novel 5HT3 receptor-IGF1 mechanism distinct from SSRI-induced antidepressant effects. Mol. Psychiatry.

[66] van Berlo D., Woutersen M., Muller A., Pronk M., Vriend J., Hakkert B. (2022). 10% Body weight (gain) change as criterion for the maximum tolerated dose: A critical analysis. Regul. Toxicol. Pharmacol..

[67] Paxinos G., Watson C. (2007). The Rat Brain in Stereotaxic Coordinates.

[68] Malberg J.E., Platt B., Sukoff Rizzo S.J., Ring R.H., Lucki I., Schechter L.E., Rosenzweig-Lipson S. (2007). Increasing the levels of insulin-like growth factor-1 by an IGF binding protein inhibitor produces anxiolytic and antidepressant-like effects. Neuropsychopharmacology.

[69] Basta-Kaim A., Szczesny E., Glombik K., Stachowicz K., Slusarczyk J., Nalepa I., Zelek-Molik A., Rafa-Zablocka K., Budziszewska B., Kubera M. (2014). Prenatal stress affects insulin-like growth factor-1 (IGF-1) level and IGF-1 receptor phosphorylation in the brain of adult rats. Eur. Neuropsychopharmacol..

[70] Schnieder T.P., Trenceska I., Rosoklia G., Stankov A., Mann J.J., Smiley J., Dwork A.J. (2014). Microglia of prefrontal white matter in suicide. J. Neuropathol. Exp..

