# Supplementary figures and images for: Central Insulin-like Growth Factor-1 Treatment Enhances Working and Reference Memory by Reducing Neuroinflammation and Amyloid Beta Deposition in a Rat Model of Sporadic Alzheimer’s Disease
Source: Pharmaceuticals (Basel). 2025 Apr 4;18(4):527. doi: 10.3390/ph18040527 (PMC12030085; doi:10.3390/ph18040527)

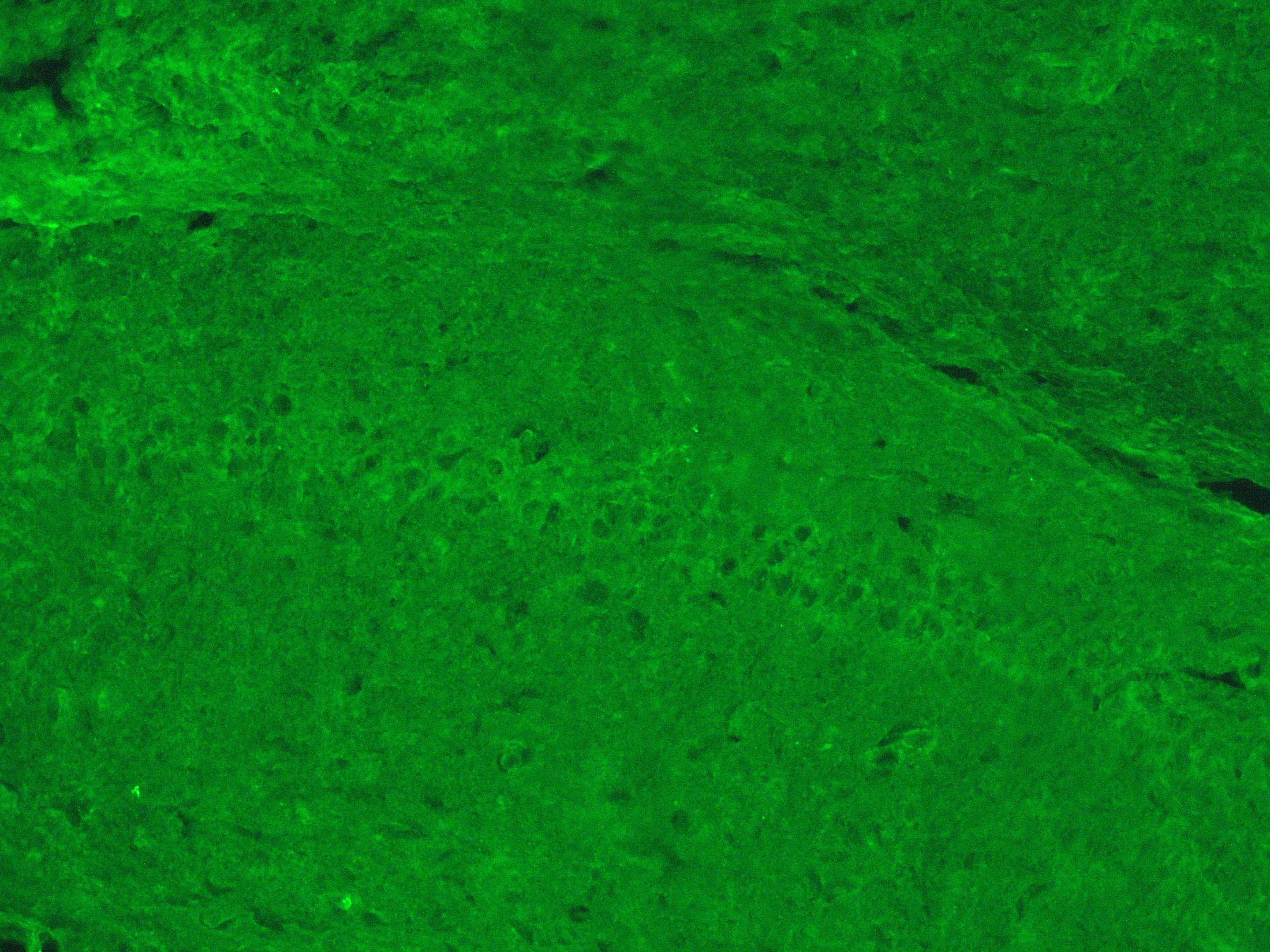

Supplement: Supplementary file 1 [file pharmaceuticals-18-00527-s001.zip › Microscope image S3Bd (VEHIGF-1).tif]

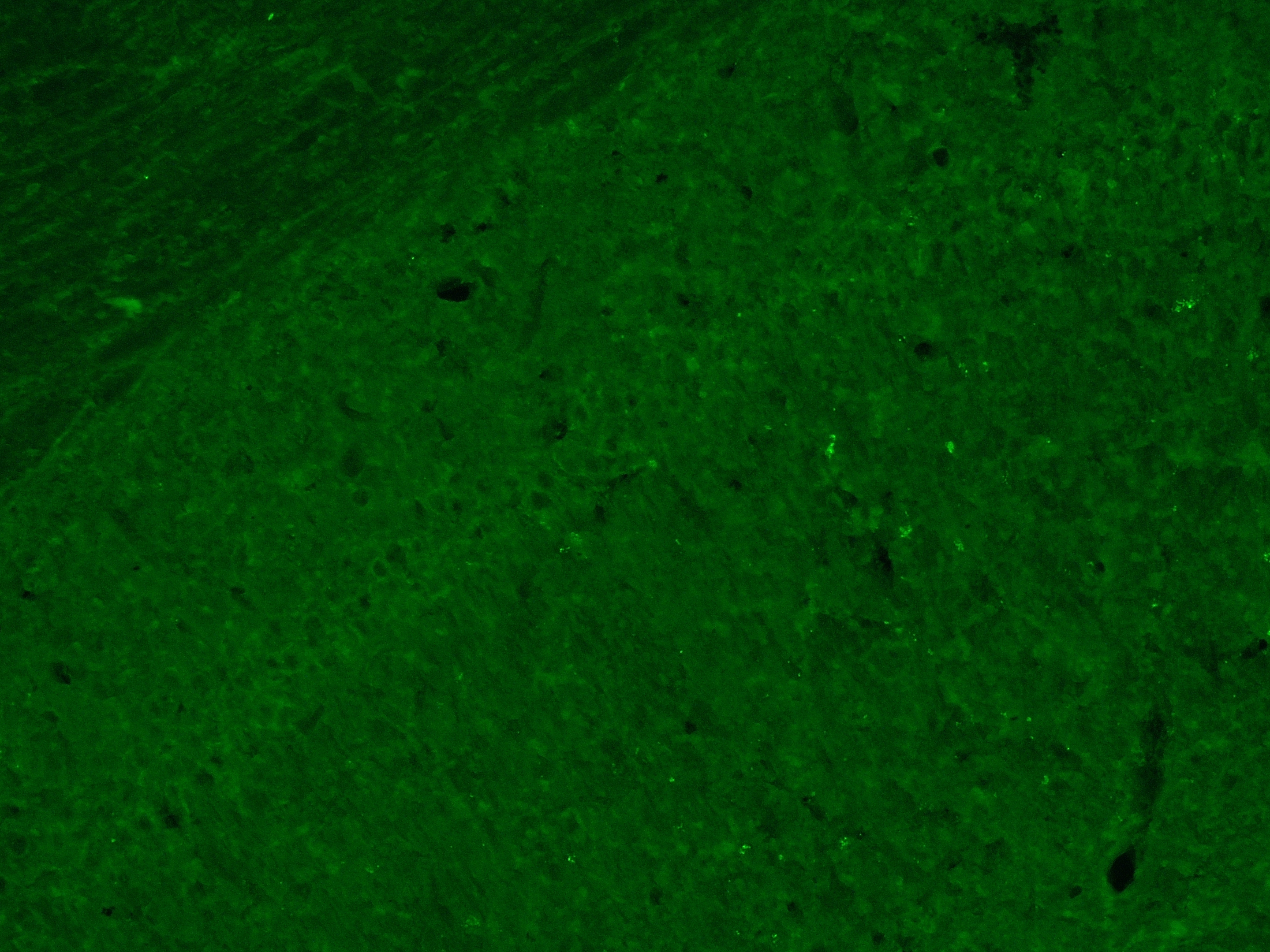

Supplement: Supplementary file 1 [file pharmaceuticals-18-00527-s001.zip › Microscope image S3Bb (STZIGF-1).tif]

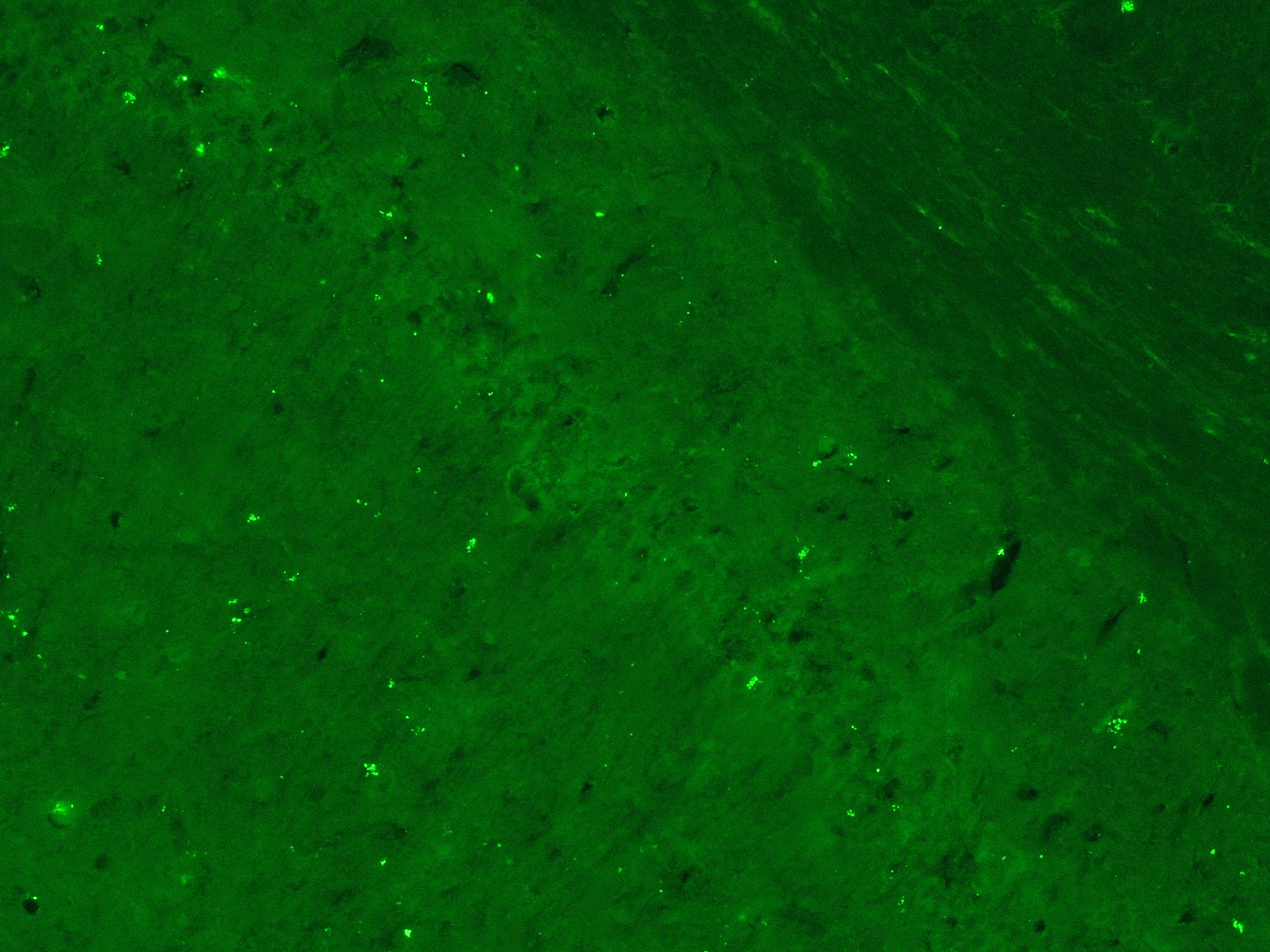

Supplement: Supplementary file 1 [file pharmaceuticals-18-00527-s001.zip › Microscope image S3Ba (STZSAL).tif]

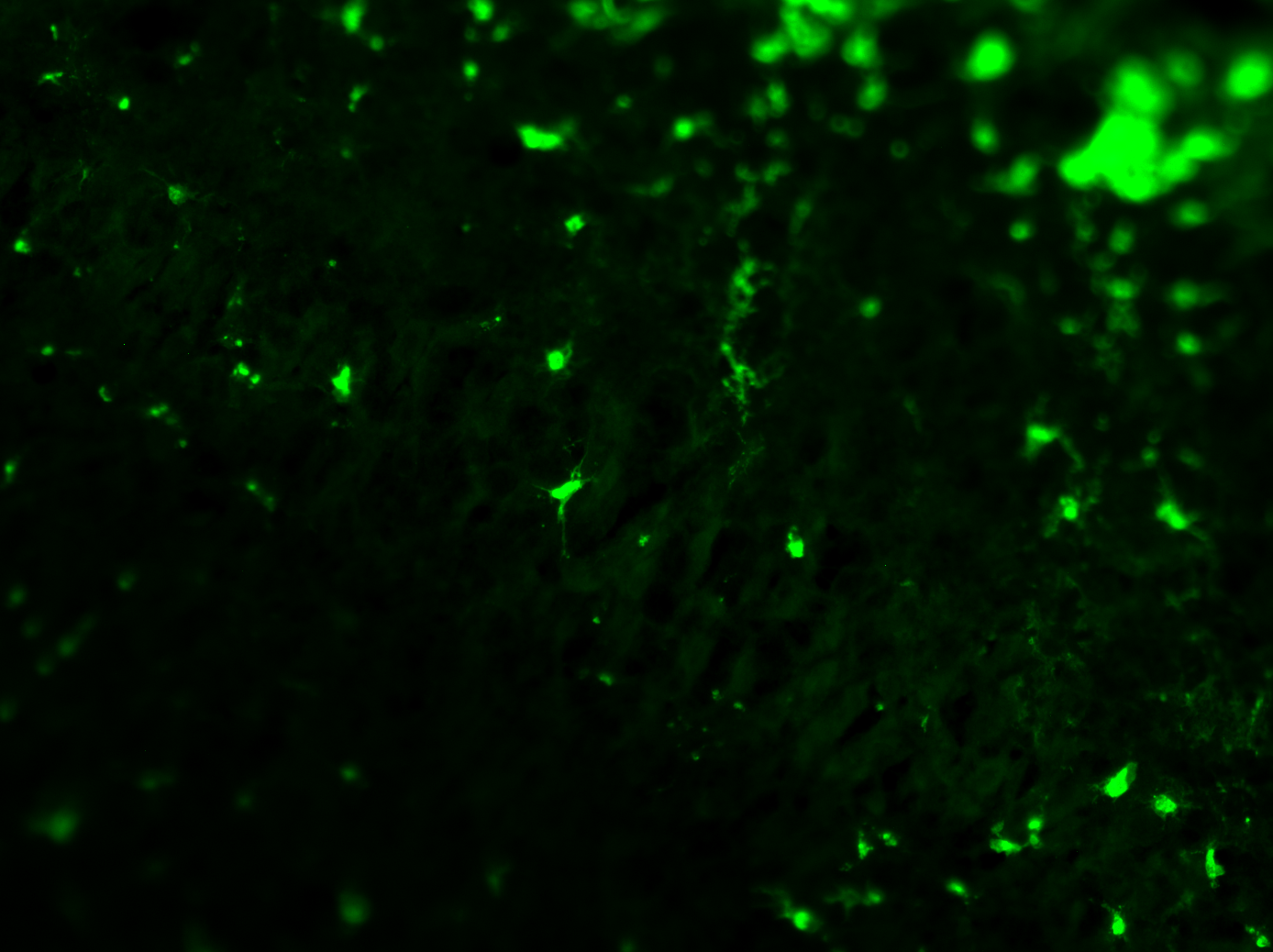

Supplement: Supplementary file 1 [file pharmaceuticals-18-00527-s001.zip › Microscope image S3Aa (STZSAL).tif]

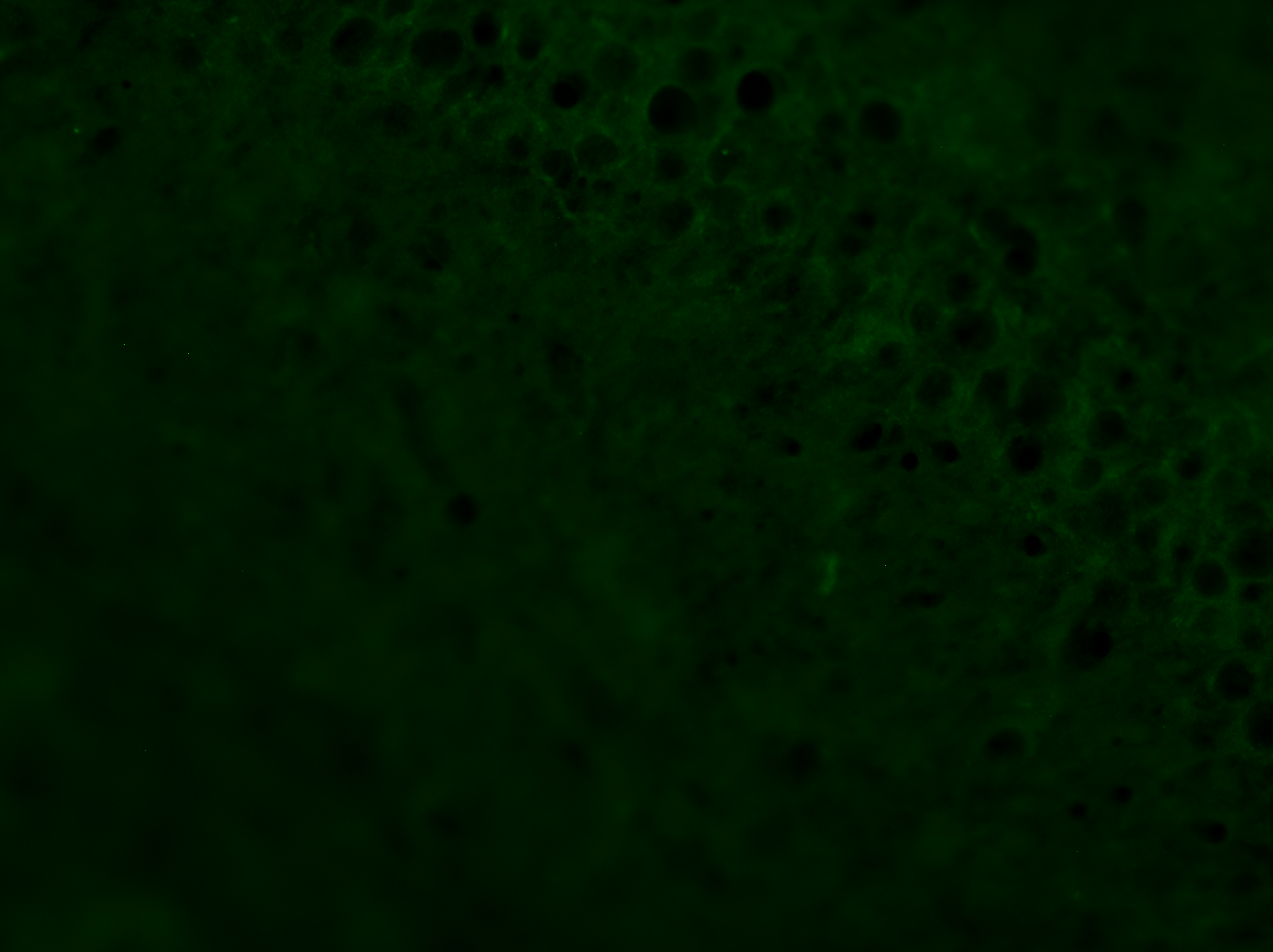

Supplement: Supplementary file 1 [file pharmaceuticals-18-00527-s001.zip › Microscope image S3Ac (VEHSAL).tif]

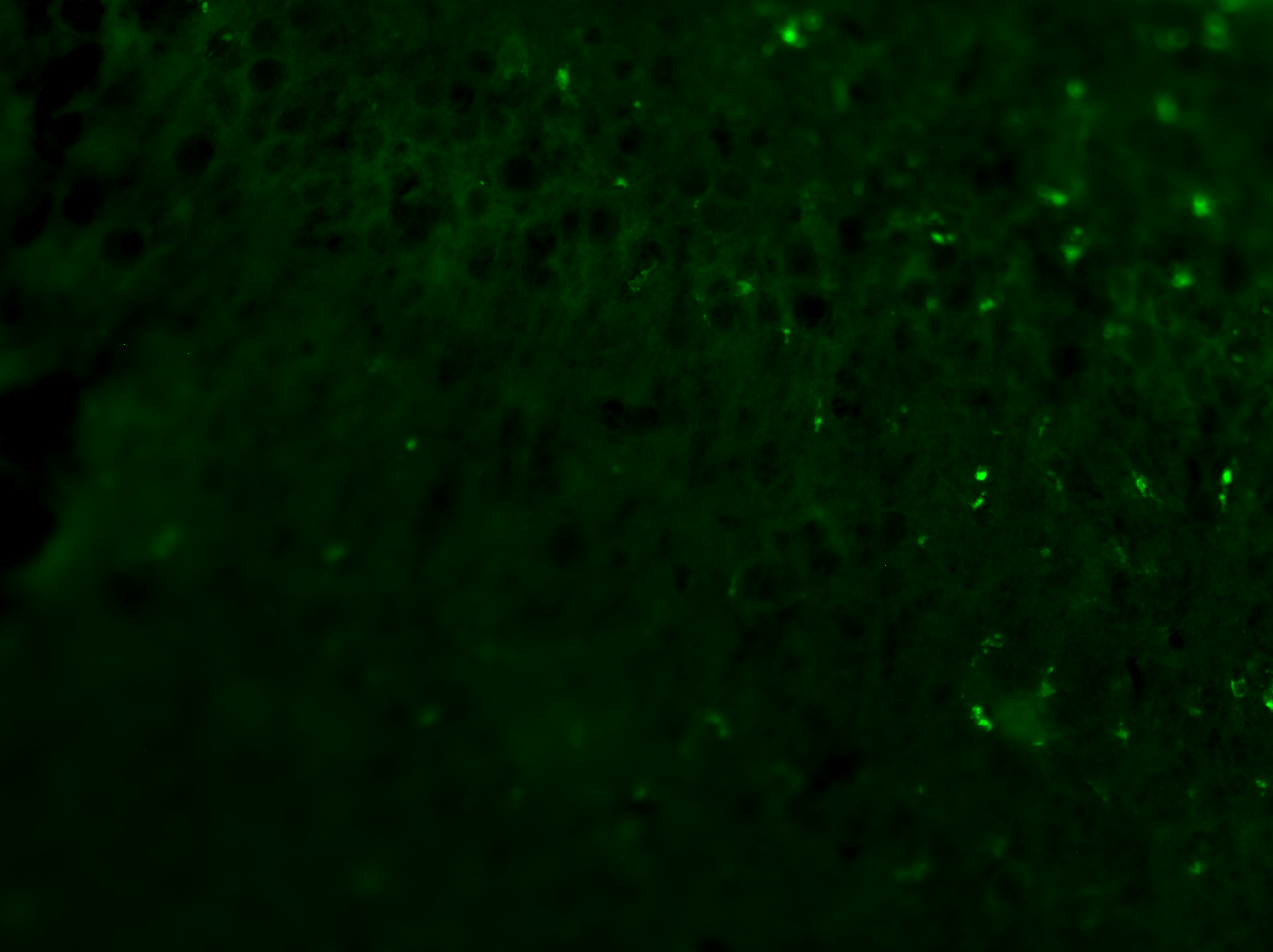

Supplement: Supplementary file 1 [file pharmaceuticals-18-00527-s001.zip › Microscope image S3Ab (STZIGF-1).tif]

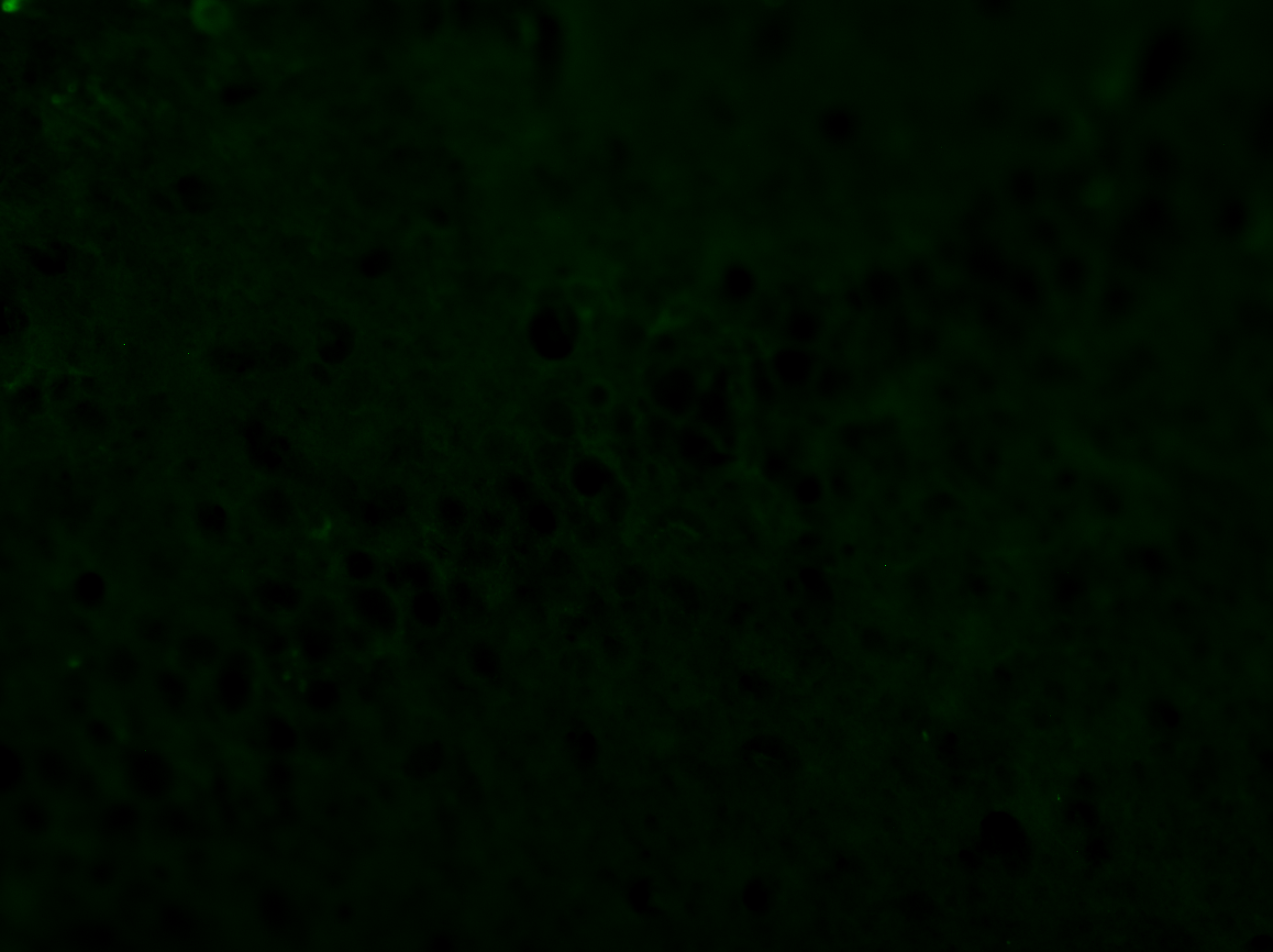

Supplement: Supplementary file 1 [file pharmaceuticals-18-00527-s001.zip › Microscope image S3Ad (VEHIGF-1).tif]

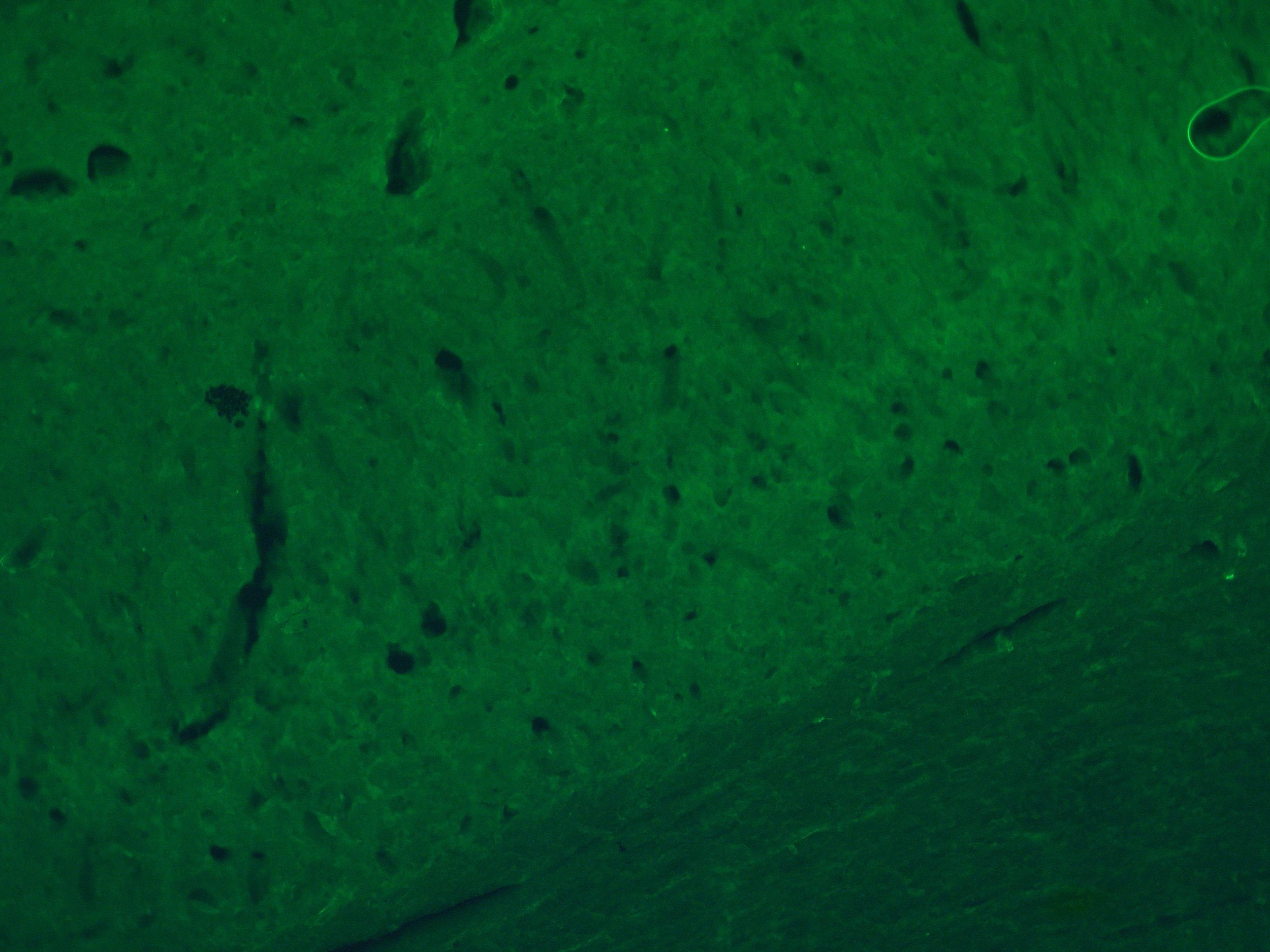

Supplement: Supplementary file 1 [file pharmaceuticals-18-00527-s001.zip › Microscope image S3Bc (VEHSAL).tif]
